# Muco‐Penetrating Lipid Nanoparticles Having a Liquid Core for Enhanced Intranasal mRNA Delivery

**DOI:** 10.1002/advs.202407383

**Published:** 2025-01-30

**Authors:** Nipuni Maniyamgama, Ki Hyun Bae, Zi Wei Chang, Jialing Lee, Melgious J. Y. Ang, Yong Jie Tan, Lisa F. P. Ng, Laurent Renia, Kevin P. White, Yi Yan Yang

**Affiliations:** ^1^ Bioprocessing Technology Institute (BTI) Agency for Science, Technology and Research (A*STAR) 20 Biopolis Way, Centros #06‐01 Singapore 138668 Republic of Singapore; ^2^ A*STAR Infectious Diseases Labs (A*STAR ID Labs) Agency for Science, Technology and Research (A*STAR) 8A Biomedical Grove, Immunos #05‐13 Singapore 138648 Republic of Singapore; ^3^ Lee Kong Chian School of Medicine Nanyang Technological University Singapore 138648 Republic of Singapore; ^4^ School of Biological Sciences Nanyang Technological University Singapore 138648 Republic of Singapore; ^5^ Department of Biochemistry and Precision Medicine Translational Research Program Yong Loo Lin School of Medicine National University of Singapore Singapore 119228 Republic of Singapore

**Keywords:** intranasal, ionizable, liquid lipid nanoparticles, mRNA, muco‐penetrating

## Abstract

Intranasal delivery of mRNA vaccines offers promising opportunities to combat airborne viruses like SARS‐CoV‐2 by provoking mucosal immunity, which not only defends against respiratory infection but also prevents contagious transmission. However, the development of nasal mRNA vaccines has been hampered by the lack of effective means to overcome the mucus barrier. Herein, ionizable lipid‐incorporated liquid lipid nanoparticles (iLLNs) capable of delivering mRNA cargo across airway mucosa are designed. Adjusting the ratios of ionizable and cationic lipids allows fine‐tuning of the p*K*_a_ of iLLNs to the range of nasal mucosal pH (5.5–6.5), thus facilitating mucus penetration via the formation of near‐neutral, PEGylated muco‐inert surfaces. When nasally administered to mice, the top candidate iLLN‐2/mRNA complexes enable about 60‐fold greater reporter gene expression in the nasal cavity, compared to the benchmark mRNA‐lipid nanoparticles (ALC‐LNP) having the same lipid composition as that of BNT162b2 vaccine. Moreover, a prime‐boost intranasal immunization of iLLN‐2/mRNA complexes elicits a greater magnitude of SARS‐CoV‐2 spike‐specific mucosal IgA and IgG response than ALC‐LNP, without triggering any noticeable inflammatory reactions. Taken together, these results provide useful insights for the design of nasally deliverable mRNA formulations for prophylactic applications.

## Graphical abstract

Muco‐penetrating, ionizable lipid‐incorporated liquid lipid nanoparticles (iLLNs) with tunable p*K*_a_ values are designed for enhanced intranasal mRNA delivery. The top candidate iLLN‐2/mRNA complexes demonstrate superior transgene expression in the nasal cavity and antigen‐specific mucosal immunity in mice over the benchmark mRNA‐lipid nanoparticles while avoiding harmful proinflammatory responses, showing promise as a safer and more effective nasal mRNA formulation.

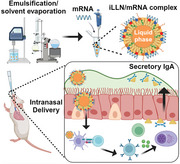

## 1 Introduction

The global outbreak of COVID‐19 has sparked an unprecedented development of lipid nanoparticle (LNP)‐formulated mRNA vaccines to combat SARS‐CoV‐2, such as BNT162b2 (Pfizer‐BioNTech) and mRNA‐1273 (Moderna).^[^ ^1^ ^]^ Currently, most COVID‐19 mRNA vaccines in use or under clinical trials are intended for intramuscular administration, which predominantly evokes systemic immunity but not mucosal immunity. The latter is critical for preventing airway infection and onward transmission.^[^ ^2^ ^]^ mRNA‐vaccinated individuals were found to acquire significantly weaker neutralizing activity in the respiratory mucosa compared with that in the blood, even after two doses of either BNT162b2 or mRNA‐1273.^[^ ^3^ ^]^ Compared with intramuscular injection, intranasal vaccination offers distinct advantages, such as non‐invasiveness, high patient compliance, and the ability to boost protective immunity in the upper respiratory tract, which represents the primary route of entry of SARS‐CoV‐2 and other viruses.^[^ ^4^ ^]^ However, the development of nasal mRNA vaccines remains a formidable challenge, largely due to the sticky mucus layer that traps foreign particulates and facilitates their removal via the mucociliary clearance machinery.^[^ ^5^ ^]^

Extensive efforts have been devoted to engineering nanoparticles with muco‐penetrating properties. One commonly used approach is to impart “stealth” properties to nanoparticles through muco‐inert polymeric coatings.^[^ ^6^ ^]^ Since mucin fibers possess both negatively charged and hydrophobic domains, hydrophilic non‐ionic polymers, such as poly(ethylene glycol) (PEG), poly(2‐alkyl‐2‐oxazolines), and poly(vinyl alcohol), have been exploited to shield the particle surface from adhesive interactions with mucus.^[^ ^7^ ^]^ For example, dense PEG‐coated polymeric nanoparticles exhibited rapid airway mucus penetration and improved pulmonary delivery of corticosteroids.^[^ ^8^ ^]^ However, the need for a relatively high PEG coverage (> 5% by weight) may pose a disadvantage to mRNA‐LNPs, which require the adsorption of serum proteins for their receptor‐mediated endocytosis.^[^ ^9^ ^]^ Accumulating evidence reveals that increasing the PEG‐lipid density up to ≈5 mol% can promote mucus permeability of mRNA‐LNPs, but negatively impacts their transfection efficiency in the respiratory tract.^[^ ^10^ ^]^ In a recent mouse study, no significant IgG response was induced by nasally administered mRNA‐LNPs carrying 2 mol% PEG‐lipid, highlighting the need for alternative approaches for effective mucosal mRNA delivery.^[^ ^11^ ^]^

Liquid lipid nanoparticles (LLNs), which consist of a liquid lipid core stabilized by a shell of amphiphilic lipids, have gained increasing attention as promising colloidal drug carriers, due to the ease of manufacture, enhanced drug loading capacity, and high physical stability.^[^ ^12^ ^]^ In contrast to traditional solid lipid nanoparticles having a crystalline solid core, the core of LLNs is composed of lipids with low melting temperatures, which exist in a liquid state at body temperature. Multiple studies have reported that the rigidity of nanoparticles can be altered to facilitate their mucus permeability.^[^ ^13^ ^]^ Owing to the more flexible structure, soft nanomaterials were found to move faster through mucus network than stiff ones.^[^ ^14^ ^]^ More recently, the generation of a liquid oil core has been shown to augment the muco‐penetrating ability of nanoparticles by rendering them with high deformability to enable easier movement in the mucus.^[^ ^15^ ^]^ In this perspective, the liquid lipid core of LLNs makes them an attractive candidate for the development of nasal mRNA vaccine vehicles capable of bypassing the mucus barrier.

Herein, we report an approach to boost intranasal mRNA delivery through fine‐tuning the acid dissociation constant (p*K*_a_) of muco‐penetrating, ionizable lipid‐incorporated LLNs (iLLNs). Ionizable lipids bearing a tertiary amine headgroup, such as ALC‐0315 and SM‐102 used in the formulation of BNT162b2 and mRNA‐1273, respectively, have been considered a key element of mRNA‐LNP vaccines.^[^ ^16^ ^]^ Upon entering the acidic endosomal milieu (pH < 6.5), the tertiary amine group of ionizable lipids becomes protonated and associates with the anionic endosomal phospholipids, thereby favoring the membrane fusion and mRNA release into the cytoplasm.^[^ ^17^ ^]^ We hypothesize that, by mixing an ionizable lipid with a cationic lipid at an optimal ratio, it would be possible to formulate iLLNs exhibiting p*K*_a_ values in the range of nasal mucosal pH (5.5–6.5).^[^ ^18^ ^]^ Following nasal administration, iLLNs are expected to form electroneutral, PEGylated “muco‐inert” surfaces that can maximally avoid adhesive interactions with mucin, while efficiently crossing the mucus layer due to the existence of a liquid core, providing high deformability.^[^ ^19^ ^]^ Once endocytosed, cationic charges on iLLNs will increase as the pH decreases below their p*K*_a_ values, thus promoting endosomal evasion and cytosolic mRNA release. To test this hypothesis, we formulated a series of iLLNs by mixing the ionizable lipid ALC‐0315 with the cationic lipid DOTMA at varying weight ratios and investigated their nasal mRNA delivery efficiencies. When nasally administered to mice, the leading candidate iLLN‐2/mRNA complexes achieved substantially higher gene expression in the upper respiratory tract than its cationic counterpart and ALC‐0315‐based mRNA‐LNP (ALC‐LNP). Moreover, a two‐dose intranasal vaccination with iLLN‐2/mRNA complexes but not ALC‐LNP produced an evident increase of anti‐SARS‐CoV‐2 spike IgA and IgG responses in the nasal mucosa. Unlike ALC‐LNP with poor tolerability, iLLN‐2/mRNA complexes exhibited minimal levels of local and systemic inflammatory reactions, showing promise as a safer and more effective intranasal mRNA formulation.

## 2 Results

### 2.1 Design and Characterization of p*K*_a_‐Tunable iLLN/mRNA Complexes

**Figure 1:**
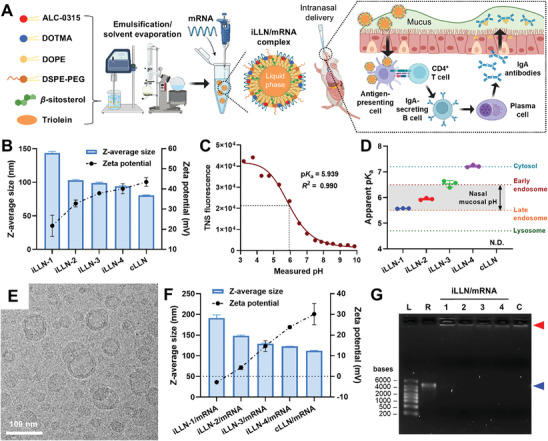
Preparation and characterization of iLLN/mRNA complexes for intranasal mRNA delivery. A) Scheme illustrating the formation of an iLLN/mRNA complex via an emulsification/solvent evaporation technique and its plausible pathway for eliciting secretory IgA response in the nasal mucosa. Created with BioRender.com. B) Z‐average size and zeta potential of bare iLLN and cLLN formulations in nuclease‐free water. Mean ± SD (*n* = 3). C) Typical TNS fluorescence titration curve of iLLN‐2. p*K*_a_ was determined as the pH at half‐maximal fluorescence intensity. D) Apparent p*K*_a_ values of iLLN and cLLN formulations. The gray region indicates the range of nasal mucosal pH (5.5–6.5). N.D.: not detectable. Mean ± SD (*n* = 3). E) Representative cryo‐TEM image of iLLN‐2. Scale bar: 100 nm. F) Z‐average size and zeta potential of iLLN/mRNA and cLLN/mRNA complexes formulated with PVX1010 mRNA. The measurements were conducted in pH 6 saline. Mean ± SD (*n* = 3). G) Gel electrophoresis analysis of iLLN/mRNA and cLLN/mRNA complexes incubated in pH 6 saline for 1 h. L: ladder, R: free mRNA, 1–4: iLLN‐1/mRNA to iLLN‐4/mRNA complexes, C: cLLN/mRNA complex. The blue and red arrowheads indicate the location of free mRNA and its corresponding complexes, respectively.

**Figure** **1**A illustrates the construction of iLLNs via the self‐assembly of lipid components (ALC‐0315, DOTMA, *β*‐sitosterol, DOPE, triolein, and DSPE‐PEG) using an emulsification/solvent evaporation technique. DOTMA, a cationic lipid containing a quaternary ammonium headgroup, has broadly been applied to form lipoplexes for systemic delivery of mRNA.^[^ ^20^ ^]^ *β*‐sitosterol is a plant‐derived cholesterol analog that has been shown to improve mRNA translation efficiency of LNPs via modulation of the endocytic recycling machinery.^[^ ^21^ ^]^ Replacing cholesterol with *β*‐sitosterol is anticipated to avoid the recognition by cholesterol transporters residing on endosomal membranes, which can cause exocytosis of LNPs and thus reduce their cellular retention.^[^ ^22^ ^]^ DOPE was incorporated as a fusogenic lipid to destabilize the endosomal membrane by facilitating the formation of a non‐bilayer hexagonal H_II_ phase at acidic pH.^[^ ^23^ ^]^ Triolein, a naturally occurring triglyceride, was added to form a liquid lipid core at physiological temperature due to its low melting temperature (5.53 °C).^[^ ^24^ ^]^ DSPE‐PEG was chosen to PEGylate the surface of iLLNs to render them with colloidal stability and mucus‐penetrating ability.^[^ ^25^ ^]^ To formulate iLLNs, all lipids with specified weight ratios were dissolved in chloroform/ethanol mixture, followed by dispersion into nuclease‐free water and ultrasonication to form an oil‐in‐water (O/W) nanoemulsion. Upon evaporation of the solvents, hydrophobic interactions among the lipids promote spontaneous self‐assembly of iLLNs having a triolein‐filled core stabilized by an amphiphilic lipid shell. A simple mixing of iLLNs with mRNA would lead to the formation of nanosized complexes via electrostatic interactions. We hypothesize that, upon intranasal administration, optimally p*K*_a_‐tuned iLLN/mRNA complexes could traverse the mucosal barrier and transfect antigen‐presenting cells (APCs), which can present antigens to CD4^+^ T cells and induce IgA‐secreting B cell development for mucosal immunity.^[^ ^26^ ^]^

In this study, a series of iLLNs were synthesized by varying the weight ratio of ALC‐0315 to DOTMA (Table 1). A pure ALC‐0315‐based formulation termed iLLN‐1 was generated with the addition of ALC‐0315 at 48 wt% (i.e., 48% weight percentage of the total lipids). The ALC‐0315/DOTMA composite‐based formulations termed iLLN‐2 to iLLN‐4 were produced by adjusting the ALC‐0315:DOTMA ratio from 3:1 to 1:3, while keeping the weight ratio among the other four components constant (DOPE/*β*‐sitosterol/triolein/DSPE‐PEG = 27/20/3/2). For comparison with iLLNs, a cationic LLN (cLLN) formulation was prepared by replacing ALC‐0315 in the formula of iLLN‐1 with an equal weight percentage (48 wt%) of DOTMA. Increasing the weight percentage of DOTMA led to a gradual rise in the zeta potential of iLLNs with a concomitant reduction in their Z‐average size (Figure 1B). All iLLN formulations had a monodispersed size distribution as indicated by the low polydispersity index of < 0.3 (Figure S1, Supporting Information).^[^ ^27^ ^]^

**Table 1:** Lipid composition of a series of iLLN and cLLN formulations.

Formulations	Weight percentage [%]					
	ALC‐0315	DOTMA	DOPE	*β*‐sitosterol	Triolein	DSPE‐PEG
iLLN‐1	48	0	27	20	3	2
iLLN‐2	36	12	27	20	3	2
iLLN‐3	24	24	27	20	3	2
iLLN‐4	12	36	27	20	3	2
cLLN	0	48	27	20	3	2

The surface ionization behavior of iLLNs was examined by measuring their p*K*_a_ values using in situ TNS fluorescence titration (Figure 1C). In the case of iLLNs, TNS fluorescence escalated steeply as the pH decreased below the p*K*_a_, indicative of the protonation of ionizable amine groups at acidic pH.^[^ ^28^ ^]^ In contrast, cLLN lacking an ionizable lipid did not exhibit a sharp pH transition in the TNS fluorescence curve (Figure S2, Supporting Information). As shown in Figure 1D, the p*K*_a_ of iLLNs could be tuned from ca. 5.57 to 7.22 by increasing the weight percentage of DOTMA from 0 to 36 wt%. iLLN‐1 to iLLN‐3 were found to have p*K*_a_ values in the range of early endosomal pH (5.5–6.5),^[^ ^29^ ^]^ suggesting their potential ability to escape from early endosomes for cytosolic delivery of mRNA cargo. Considering the average pH value of nasal mucosa (≈6),^[^ ^18^ ^]^ iLLN‐2 with a p*K*_a_ of 5.94 was considered ideal for mucus penetration because this formulation would form nearly neutral surfaces to minimize adhesive interactions with mucin. Cryo‐TEM elucidated the spherical capsule‐like structure of iLLN‐2, which could arise from the existence of triolein‐rich core (Figure 1E). This morphology was distinguishable from the characteristic “bleb” structure commonly observed in mRNA‐LNPs.^[^ ^1^ ^]^ Z‐average size and zeta potential of iLLN/mRNA complexes were examined in pH 6 saline to simulate the acidic nasal environment (Figure 1F). As expected, mRNA complexation caused an increase in the particle size with an accompanying reduction in the surface charge. Notably, iLLN‐2/mRNA complexes exhibited a near‐neutral surface charge (≈4.26 mV), favorable for mucus permeation by avoiding adhesive entrapment.^[^ ^7^ ^]^ Gel electrophoresis assay showed complete retardation of mRNA migration upon complexation with all iLLNs, indicating full mRNA condensation (Figure S3, Supporting Information). No leakage of free mRNA was observed from all iLLN/mRNA complexes incubated in pH 6 saline, confirming their stability under the acidic nasal condition (Figure 1G).

### 2.2 Enhanced Transfection Potency of iLLN/mRNA Complexes

**Figure 2:**
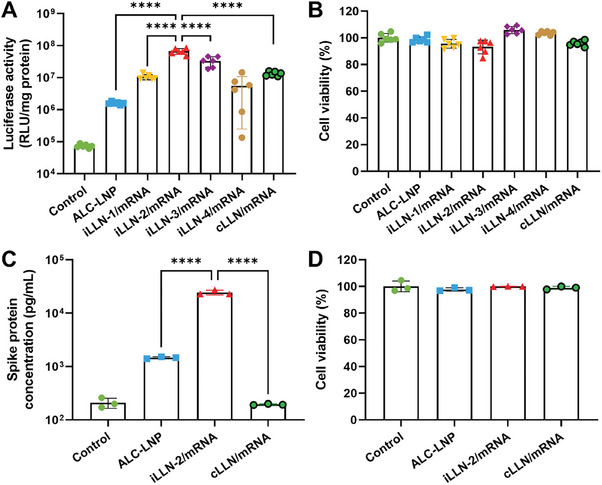
In vitro transfection potency of iLLN/mRNA complexes. A) Luciferase expression level and B) viability of A549 cells treated for 48 h with ALC‐LNP, iLLN/mRNA, or cLLN/mRNA complexes formulated with FLuc mRNA. Mean ± SD (*n* = 6); ^****^ *p* < 0.0001 (one‐way ANOVA with Tukey's post hoc test). C) SARS‐CoV‐2 Delta variant spike protein expression and D) viability of A549 cells treated for 48 h with ALC‐LNP, iLLN‐2/mRNA, or cLLN/mRNA complexes formulated with PVX1010 mRNA. Mean ± SD (*n* = 3); ^****^ *p* < 0.0001 (one‐way ANOVA with Tukey's post hoc test).

We assessed the translation efficacy and cytotoxicity of iLLN/mRNA complexes formulated with a firefly luciferase reporter‐encoding mRNA (FLuc mRNA) on two different cell lines: A549 and DC2.4. A549 is a well‐studied lung cell line having phenotypic similarity to human alveolar epithelial cells,^[^ ^30^ ^]^ while DC2.4 is a dendritic cell line widely used as a model for APCs, which play a pivotal role in the initiation of adaptive immunity.^[^ ^31^ ^]^ Interestingly, the weight ratio of ALC‐0315 to DOTMA had a profound influence on mRNA translation efficiency of iLLNs (**Figure** **2**A). For instance, FLuc expression levels escalated when the weight percentage of DOTMA was raised from 0 to 12 wt%. A further increase in the DOTMA fraction caused a reduction in FLuc mRNA expression. Notably, iLLN‐2 formed at the ALC‐0315:DOTMA ratio of 3:1 exhibited significantly (*P* < 0.0001) greater mRNA translation potency compared to iLLN‐1 bearing ALC‐0315 alone and cLLN bearing DOTMA alone. Encouragingly, iLLN‐2 was much more efficacious in inducing mRNA translation than the benchmark ALC‐LNP made of the same lipid composition used in BNT162b2 vaccine.^[^ ^1^ ^]^ All iLLN formulations were non‐toxic to A549 cells, implying that variation of ALC‐0315:DOTMA ratios did not adversely impact the viability of the transfected cells (Figure 2B). The superior translation efficacy of iLLN‐2/mRNA complexes was also verified in DC2.4 dendritic cells (Figure S4, Supporting Information). Based on these findings, it was inferred that a combination of ALC‐0315 and DOTMA at an optimal ratio might drive an enhancement of the intracellular mRNA delivery performance of iLLNs.

Next, we examined the applicability of the best‐performing iLLN‐2 for delivery of a codon‐optimized mRNA encoding SARS‐CoV‐2 Delta variant spike protein (PVX1010 mRNA).^[^ ^32^ ^]^ It has been reported that the protein expression efficiencies of LNPs can vary depending on the lengths of mRNA cargos.^[^ ^33^ ^]^ Of note, PVX1010 mRNA has a much larger size (4047 nucleotides) than FLuc mRNA (1922 nucleotides). As depicted in Figure 2C, a remarkably higher concentration (≈24336 pg mL^−1^) of SARS‐CoV‐2 spike protein was detected in the supernatant of A549 cells treated with iLLN‐2/mRNA complexes than those with ALC‐LNP (≈1458 pg mL^−1^) and with cLLN/mRNA complexes (≈194 pg mL^−1^). Negligible cytotoxicity was observed for all the tested formulations (Figure 2D). These results demonstrated the capability of iLLN‐2 to intracellularly deliver mRNA cargos of different sizes with minimal cytotoxicity.

### 2.3 pH‐Dependent Membrane‐Destabilizing Activity of iLLN/mRNA Complexes

**Figure 3:**
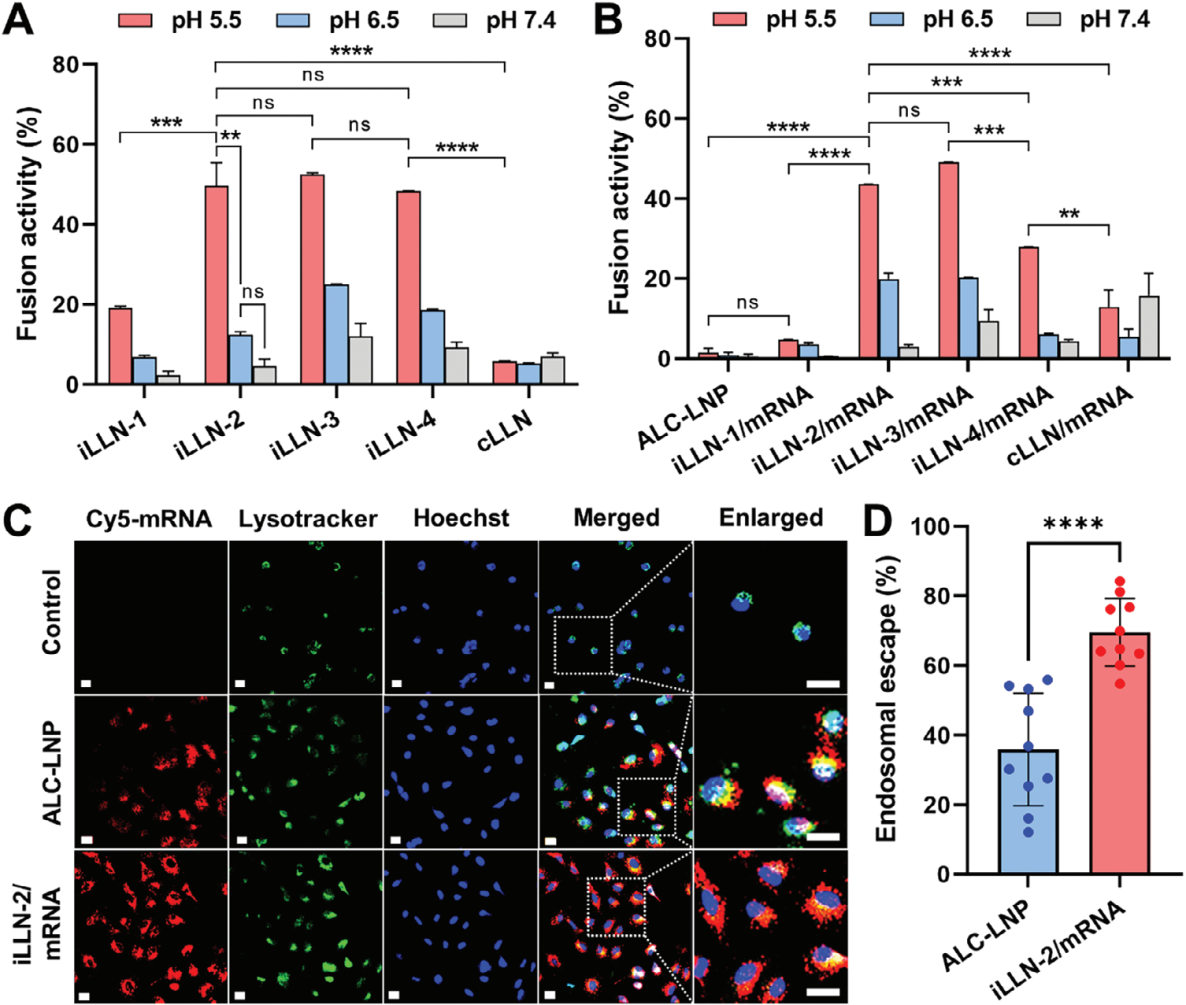
pH‐dependent membrane‐destabilizing activity of iLLNs and iLLN/mRNA complexes. Fusion activity of A) bare iLLN and cLLN formulations or B) ALC‐LNP, iLLN/mRNA, or cLLN/mRNA complexes at three different pH values (5.5, 6.5, or 7.4). Mean ± SD (*n* = 3); ^****^ *p* < 0.0001; ^***^ *p* < 0.001; ^**^ *p* < 0.01; ns: nonsignificant (one‐way ANOVA with Tukey's post hoc test). C) Representative confocal microscopic images of A549 cells taken at 4 h after transfection of ALC‐LNP or iLLN/mRNA complexes formulated with Cy5‐mRNA. Green and blue fluorescent regions show the location of endo‐lysosomes and nuclei, respectively. Scale bars: 20 µm. D) Endosomal escape efficiencies of ALC‐LNP and iLLN‐2/mRNA complexes in 10 individual cells were analyzed using ImageJ software. Mean ± SD (*n* = 10); ^****^ *p* < 0.0001 (two‐tailed unpaired Student's *t*‐test).

To understand the mechanism responsible for the superior transfection efficacy of iLLN/mRNA complexes, we first examined the ability of bare iLLNs to destabilize erythrocyte membrane under three different pH conditions simulating extracellular (pH 7.4), early endosomal (pH 6.5), and late endosomal (pH 5.5) environment.^[^ ^34^ ^]^ At pH 7.4, only marginal levels (< 15%) of membrane disruption were detected from all iLLNs (**Figure** **3**A). However, the membrane fusion activity of iLLNs gradually escalated with lowering pH from 7.4 to 5.5, possibly due to the acquisition of positive charges by protonation of the ionizable lipid at acidic pH.^[^ ^17^ ^]^ On the contrary, the fusion activity of cLLN did not increase in response to acidic pH, reflecting the lack of an ionizable amine group in DOTMA. Of note, iLLN‐2, −3 and −4 were markedly (*P* < 0.001) more effective in destabilizing erythrocyte membrane at pH 5.5 than iLLN‐1 and cLLN, suggestive of an improved fusogenic effect of ALC‐0315 and DOTMA in combination compared to either lipid alone. A similar trend was observed from iLLN/mRNA complexes, implying that the membrane‐destabilizing properties of iLLNs were not largely compromised by mRNA complexation (Figure 3B). Interestingly, iLLN‐2/mRNA complexes exhibited better fusion activity in the endosomal pH range than the conventional ALC‐LNP formulation. This feature would be beneficial for enhancing transfection potency because it allows efficient evasion of endosomal sequestration and thus favors the liberation of mRNA payload into the cytoplasm.^[^ ^16^ ^]^

We then performed a confocal laser scanning microscopic study to substantiate whether the enhanced fusogenic effect of iLLN‐2/mRNA complexes could indeed facilitate their endosomal escape. A fluorescent dye (Cy5)‐tagged mRNA was used to formulate ALC‐LNP and iLLN‐2/mRNA complexes to visualize the subcellular distribution, whereas the endo‐lysosomes and nuclei were stained with LysoTracker and Hoechst dye, respectively (Figure 3C). Most of ALC‐LNP signals were co‐localized with those of endo‐lysosomes at 4 h after mRNA transfection, suggesting that ALC‐LNPs were internalized by endocytic pathway and then sequestered in endo‐lysosomal vesicles.^[^ ^35^ ^]^ However, much less co‐localization was seen between iLLN‐2/mRNA complexes and endo‐lysosomes, indicative of an increased distribution of these complexes into the cytoplasm. This observation was corroborated by a substantial rise in the endosomal escape efficiency of iLLN‐2/mRNA complexes (69.5 ± 9.2%) relative to ALC‐LNP (35.8 ± 15.3%; Figure 3D; Table S1, Supporting Information). Collectively, it was conceivable that iLLN‐2/mRNA complexes effectively promoted endosomal disruption and cytosolic mRNA release via their pH‐dependent membrane‐destabilizing activity.

### 2.4 Muco‐Penetrating Properties of iLLN/mRNA Complexes

**Figure 4:**
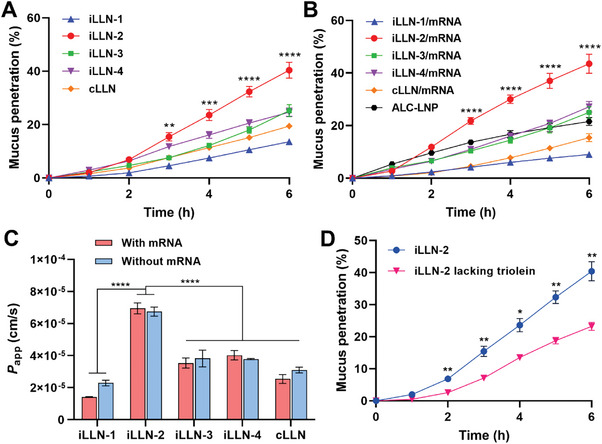
Assessment of mucus permeability of iLLNs and iLLN/mRNA complexes. The percentage of mucus penetration of A) bare iLLN and cLLN formulations or B) ALC‐LNP, iLLN/mRNA, or cLLN/mRNA complexes in the transwell mucus diffusion model at pH 6. Mean ± SD (*n* = 3); ^****^ *p* < 0.0001; ^***^ *p* < 0.001; ^**^ *p* < 0.01 versus the other groups (one‐way ANOVA with Tukey's post hoc test). C) Apparent permeability coefficient (*p*_app_) values of iLLNs, cLLN, and their corresponding mRNA complexes. Mean ± SD (*n* = 3); ^****^ *p* < 0.0001 (one‐way ANOVA with Tukey's post hoc test). D) Comparison of the mucus permeability between iLLN‐2 and iLLN‐2 lacking triolein. Mean ± SD (*n* = 3); ^**^ *p* < 0.01; ^*^ *p* < 0.05 (two‐tailed unpaired Student's *t*‐test).

Upon intranasal administration, iLLNs must cross the mucosal barrier in order to achieve successful mRNA delivery beyond the nasal epithelium.^[^ ^5^ ^]^ To this end, iLLN and cLLN formulations were fluorescently labeled with a lipophilic DiI dye to validate their muco‐penetrating ability in the transwell mucus diffusion model at pH 6 simulating the acidic nasal mucosa environment (Figure S5A, Supporting Information). DiI‐labeled formulations had Z‐average size and zeta potential comparable to those of unlabeled ones, confirming no significant impact of the fluorescent labeling on the particle structures (Figure S6, Supporting Information). iLLN‐2 was found to exert the best muco‐penetrating performance, which was superior to all the other formulations (**Figure** **4**A). The percentage of mucus penetration within 6 h followed the order of iLLN‐2 > iLLN‐3 ≈ iLLN‐4 > cLLN > iLLN‐1. Similar results were obtained with iLLN/mRNA complexes, suggesting that mRNA complexation did not largely affect the mucus transport behavior of iLLNs (Figure 4B). This finding was further supported by the quantification of apparent permeability coefficient (*P*_app_) values, which showed a close resemblance between iLLNs and their corresponding mRNA complexes (Figure 4C).

Of note, the *P*_app_ values of iLLN‐2/mRNA complexes (ca. 6.95 × 10^−5^ cm s^−1^) were remarkably higher than those of cLLN/mRNA complexes (ca. 2.54 × 10^−5^ cm s^−1^; *P* < 0.0001). The poor mucus permeability of cLLN/mRNA complexes was likely caused by their positive surface charge (30.2 ± 5.1 mV, Figure 1F), which would result in entrapment within negatively charged mucin fibers via electrostatic interactions.^[^ ^7^ ^]^ It is also interesting to note that iLLN‐2/mRNA complexes had much higher *P*_app_ values than ALC‐LNP and iLLN‐1/mRNA complexes (Figure S5B, Supporting Information) in spite of their comparable near‐neutral zeta potential (ca. 1.61 mV for iLLN‐2/mRNA; 0.27 mV for iLLN‐1/mRNA; −2.35 mV for ALC‐LNP; Figure S7A, Supporting Information). Mucus stability studies revealed a significant reduction in the derived count rate of ALC‐LNPs within 5 min after incubation in mucin‐saturated solution, suggestive of their dissociation upon contact with mucin proteins (Figure S8, Supporting Information).^[^ ^36^ ^]^ On the other hand, the derived count rate markedly increased in the case of iLLN‐1/mRNA complexes, indicating their aggregation upon contact with mucin. As expected, cLLN/mRNA complexes rapidly aggregated in the presence of mucin proteins, as evidenced by the drastic increase in the derived count rate (Figure S8, Supporting Information).^[^ ^37^ ^]^ However, iLLN‐2/mRNA complexes did not show any noticeable change in the derived count rate over 180 min, demonstrating their muco‐inert property. Based on these observations, it was conceivable that the superior muco‐inertness and near‐neutral surface charge of iLLN‐2/mRNA complexes probably contributed to their greatest mucus penetration by maximally avoiding the electrostatic interactions with mucin.

Recently, the creation of a liquid oil core has been proposed as an approach to augment the mucus permeability of nanoparticles by increasing their deformability to allow easier movement through mucus network.^[^ ^15^ ^]^ In the current study, we hypothesized that LLNs would efficiently bypass the mucus layer due to the presence of a liquid lipid core, providing high deformability. To verify this hypothesis, we compared the muco‐penetrating behavior of the top‐performing iLLN‐2 with and without triolein that serves as a component to form the liquid lipid core. Notably, iLLN‐2 demonstrated markedly higher mucus penetration than iLLN‐2 lacking triolein (Figure 4D). In this study, 3% triolein was found to be optimal because a further increase in the triolein fraction up to 12% resulted in only marginal enhancement of *P*_app_ values (Figure S9, Supporting Information). Z‐average size and zeta potential of DiI‐labeled iLLN‐2 were similar to those of its counterpart lacking triolein, suggesting that the particle size and surface charge were not the reason for the observed difference in mucus permeability (Figure S7B, Supporting Information). *P*_app_ of iLLN‐2 was greatly diminished when triolein was substituted by tristearin with a high melting temperature (72.35 °C),^[^ ^24^ ^]^ signifying the importance of a liquid lipid core in the mucus permeability (Figure S10, Supporting Information). On the contrary, the replacement of *β*‐sitosterol with cholesterol had minimal impact on the mucus permeation of iLLN‐2. These findings evinced that the liquid lipid core of iLLN‐2 plays a crucial role in governing its muco‐penetrating property.

### 2.5 Improved Intranasal mRNA Delivery with iLLN‐2/mRNA Complexes

The surface density of PEG is known to have a substantial influence on the transport of PEGylated nanoparticles across mucosal surfaces.^[^ ^8^ ^]^ Prior studies have reported that raising the PEG‐lipid density up to ≈5 mol% can improve the mucus permeability of mRNA‐LNPs, but has a negative impact on their in vivo transfection efficiency.^[^ ^10^ ^]^ In the present study, the leading candidate iLLN‐2 contains DSPE‐PEG at 2 wt%, which is equivalent to 0.5 mol% of the total lipids. To find out the optimal PEG‐lipid density for intranasal mRNA delivery, iLLN‐2/mRNA complexes with varying DSPE‐PEG contents (0.5, 1, 3, and 5 mol%) were formulated with FLuc mRNA and then administered to BALB/c mice via a nasal route. We found that an increase in DSPE‐PEG contents from 0.5 to 5 mol% led to a gradual decline in FLuc mRNA expression within the nasal cavity on both dorsal and ventral sides (Figure S11, Supporting Information). Since excessive PEGylation has shown to mitigate cellular uptake of LNPs via the formation of an anti‐biofouling surface,^[^ ^38^ ^]^ it was inferred that higher DSPE‐PEG contents probably resulted in limited internalization of iLLN‐2/mRNA complexes, thus diminishing mRNA expression levels in vivo. Based on this finding, iLLN‐2/mRNA complexes containing 0.5 mol% DSPE‐PEG were selected for further investigations.

**Figure 5:**
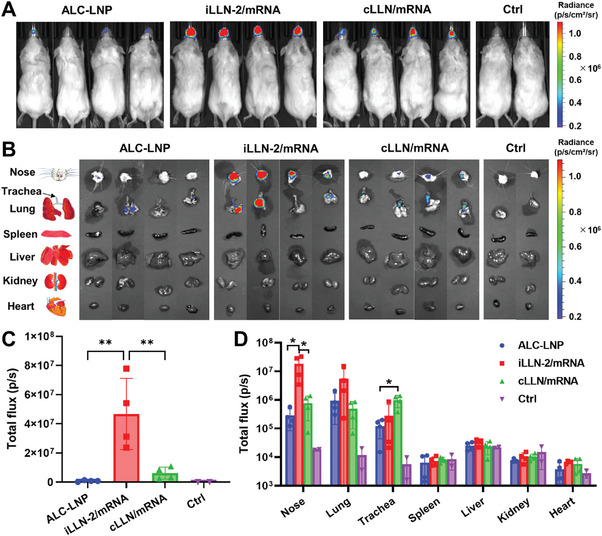
In vivo nasal mRNA delivery performance of iLLN‐2/mRNA complexes. A) Whole‐body bioluminescence images of BALB/c mice at 4 h after intranasal administration of ALC‐LNP, iLLN‐2/mRNA or cLLN/mRNA complexes (10 µg of FLuc mRNA/mouse). Naive mice injected with FLuc substrate solution were used as a mock control (Ctrl). B) Organ diagram and ex vivo luminescence images of the excised major organs. C) Total flux values of the nasal cavity measured from the whole‐body bioluminescence images. D) Total flux values of the excised organs measured from the ex vivo luminescence images. Mean ± SD (*n* = 4 for treatment groups; *n* = 2 for Ctrl); ^**^ *p* < 0.01; ^*^ *p* < 0.05 (one‐way ANOVA with Tukey's post hoc test).

Next, we compared the in vivo mRNA transfection efficiency of iLLN‐2/mRNA complexes with ALC‐LNP and cLLN/mRNA complexes following intranasal administration. At 4 h post‐administration, a strong luminescence signal was detected in the nasal cavity of the mice dosed with iLLN‐2/mRNA complexes, suggesting that these complexes traversed the nasal mucosa and facilitated mRNA delivery beyond the underlying epithelium (**Figure** **5**A). A moderate level of luminescence was observed from the mice dosed with cLLN/mRNA complexes, whereas only a faint spot was seen in the case of ALC‐LNP. This observation was supported by the total flux analysis, which showed that iLLN‐2/mRNA complexes significantly (^**^ *p* < 0.01) outperformed ALC‐LNP and cLLN/mRNA complexes (Figure 5C). To further examine biodistribution profiles, ex vivo luminescence imaging was conducted on the excised major organs (Figure 5B). Interestingly, FLuc mRNA expression was predominantly localized to the nose and lung for iLLN‐2/mRNA complexes, while the nose and trachea were mainly transfected by cLLN/mRNA complexes. The total flux values in the nose and lung were the highest for iLLN‐2/mRNA complexes, followed by cLLN/mRNA complexes and ALC‐LNP (Figure 5D). The other organs (spleen, liver, kidney, and heart) showed only marginal total flux values similar to those of the mock controls, indicating almost no mRNA expression in these organs. Overall, these results demonstrated the selective biodistribution and superior in vivo mRNA transfection performance of nasally administered iLLN‐2/mRNA complexes.

### 2.6 Induction of Mucosal Immunity by Nasally Administered iLLN‐2/mRNA Complexes

**Figure 6:**
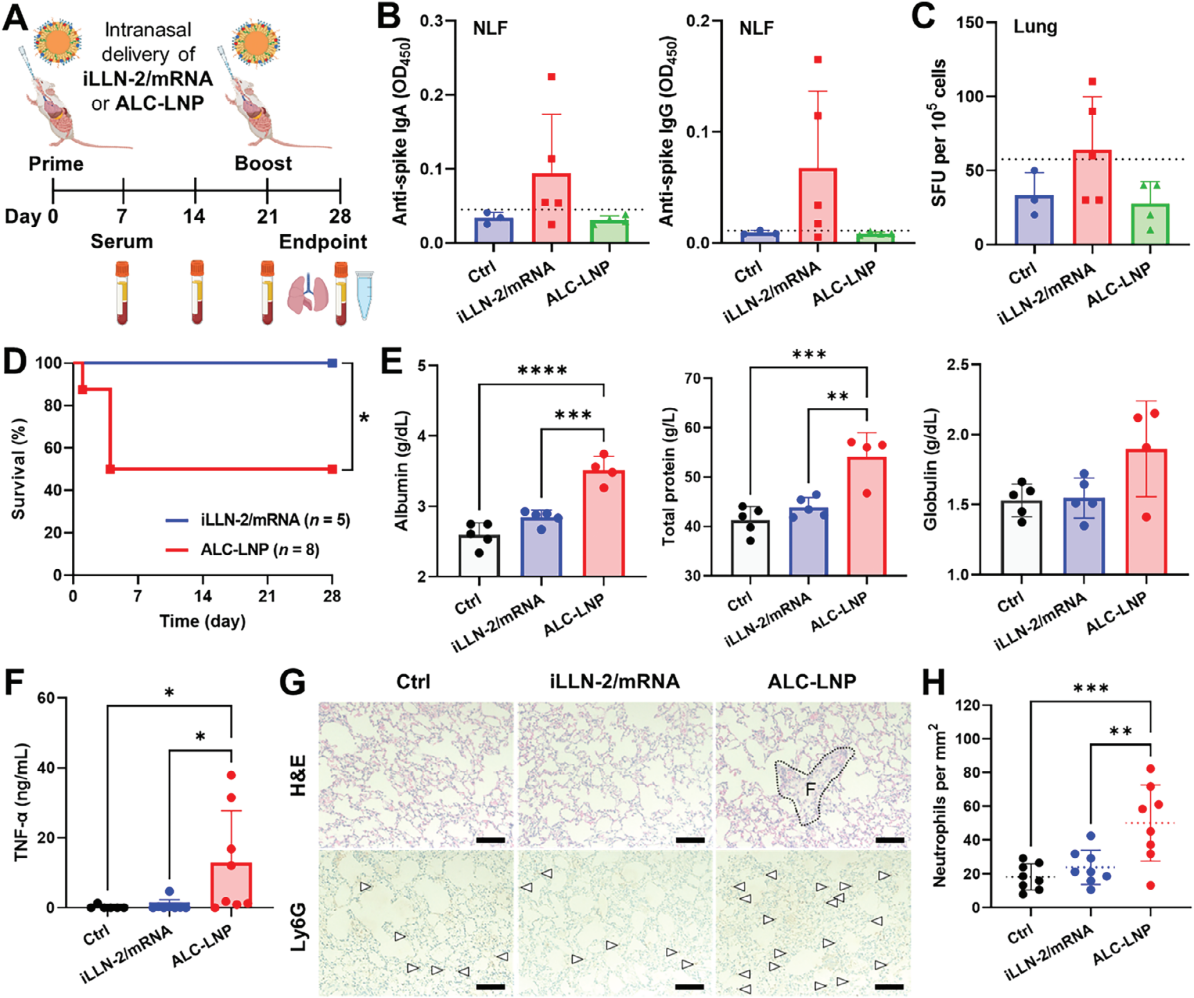
Evaluation of mucosal immunity and inflammatory reaction following intranasal administration of iLLN‐2/mRNA complexes and ALC‐LNP. A) Illustration of prime‐boost intranasal immunization and downstream assay procedures. Created with BioRender.com. B) Levels of anti‐SARS‐CoV‐2 spike IgA and IgG in NLF. The dotted line indicates the limit of detection. C) IFN‐γ‐producing spot‐forming units (SFU) per 10^5^ cells in the lung harvested on day 28. The dotted line indicates the limit of detection. Mean ± SD (*n* = 3 for Ctrl, *n* = 5 for iLLN‐2/mRNA, *n* = 4 for ALC‐LNP). D) Kaplan–Meier survival curves of the immunized mice. E) Serum levels of albumin, total protein, and globulin on day 28. Mean ± SD (*n* = 4–5); ^****^ *p* < 0.0001; ^***^ *p* < 0.001; ^**^ *p* < 0.01 (one‐way ANOVA with Tukey's post hoc test). F) Serum levels of tumor necrosis factor‐α (TNF‐α) on day 7. Mean ± SD (*n* = 8–10); ^*^ *p* < 0.05 (one‐way ANOVA with Tukey's post hoc test). G) H&E and Ly6G (neutrophil marker) staining of lung tissues harvested on day 28. Ly6G‐positive cells were marked by white arrowheads. The area labeled “F” indicates the fibrous tissue. Scale bar: 100 µm. H) Neutrophil counts in the lung were analyzed using ImageJ software. Mean ± SD (*n* = 10); ^***^ *p* < 0.001; ^**^ *p* < 0.01 (one‐way ANOVA with Tukey's post hoc test).

Having confirmed the superior nasal mRNA delivery efficiency of iLLN‐2/mRNA complexes, we attempted to investigate their potential to elicit antigen‐specific mucosal immunity. BALB/c mice were intranasally immunized on day 0 (prime) and day 21 (boost) with iLLN/mRNA complexes or ALC‐LNP at 10‐µg dose of PVX1010 mRNA/mouse (**Figure** **6**A). On day 28, we collected serum, nasal lavage fluid (NLF), and bronchoalveolar lavage fluid (BALF) to examine SARS‐CoV‐2 spike‐specific antibody responses. The prime‐boost dosing of iLLN‐2/mRNA complexes resulted in a notable increase of anti‐spike secretory IgA and IgG antibodies in NLF, while no such increase was observed in the mice dosed with ALC‐LNP (Figure 6B). This finding suggests that iLLN‐2/mRNA complexes crossed the nasal epithelium and were then engulfed by APCs, which might migrate into nose‐associated lymphoid tissues and stimulate T cells for the initiation of adaptive immune response.^[^ ^26b^ ^]^ Although ALC‐LNP and iLLN‐2/mRNA formulations stimulated the production of anti‐spike IgG antibodies in serum, both of them failed to generate IgA in BALF (Figure S12, Supporting Information), implying that nasally administered iLLN‐2/mRNA complexes provoked mucosal immunity predominantly at the upper respiratory tract. Enzyme‐linked immunospot (ELISPOT) analysis detected a relatively larger number of IFN‐γ‐producing pulmonary T lymphocytes in the mice dosed with iLLN‐2/mRNA complexes relative to ALC‐LNP, demonstrating their ability to evoke a relatively stronger antigen‐specific T cell response (Figure 6C). Recent studies have reported that PEG‐specific antibodies can be boosted by intramuscularly administered mRNA vaccines in humans.^[^ ^39^ ^]^ We sought to determine if anti‐PEG antibody generation occurred with nasally administered ALC‐LNP and iLLN‐2/mRNA formulations. On day 7 after the first administration, anti‐PEG IgM production of iLLN‐2/mRNA complexes was on average higher but not significant compared to ALC‐LNP (Figure S13, Supporting Information). Thereafter, anti‐PEG IgM titers in the iLLN‐2/mRNA group gradually dropped to levels similar to the detection limit by day 21, suggesting that the anti‐PEG IgM response was transient and eventually waned after 3 weeks post‐administration.

There is increasing evidence that mRNA‐LNPs can trigger severe proinflammatory responses and even mortality when nasally administered at high doses in mice.^[^ ^40^ ^]^ Consistently with the previous literature, intranasal administration of ALC‐LNP at 10‐µg dose caused high toxicity, resulting in the death of 50% of the dosed mice within 4 days and body weight drop by ≈8% after 7 days (Figure 6D; Figure S14, Supporting Information). In contrast, all the mice immunized with iLLN‐2/mRNA complexes survived without any obvious weight loss for 28 days. Inflammation‐related clinical chemistry parameters, such as albumin, total protein, and globulin levels, were elevated after ALC‐LNP treatment, while no noticeable changes were seen for iLLN‐2/mRNA complexes (*P* > 0.05), confirming their superior tolerability (Figure 6E; Figure S15, Supporting Information). The tendency of ALC‐LNP to cause systemic inflammation was also confirmed by a significant rise in the proinflammatory cytokine TNF‐α in the sera (Figure 6F). Furthermore, the mice dosed with ALC‐LNP showed massive infiltration of neutrophils and fibrotic tissue formation in the lung (Figure 6G). Importantly, the mice dosed with iLLN‐2/mRNA complexes had a similar number of pulmonary neutrophils as in the control group, indicative of only minimal local inflammation (Figure 6H). Taken together, the above results demonstrated that nasally administered iLLN‐2/mRNA complexes effectively induced antigen‐specific mucosal immunity without severe local and systemic inflammatory reactions.

## 3 Discussion

Delivery of mRNA vaccines via a nasal route has received enormous attention in the battle against COVID‐19 because of its potential to activate sterilizing immunity in the upper airway, the primary site of SARS‐CoV‐2 infection and onward transmission.^[^ ^4^ ^]^ However, mRNA vaccines are confronted by many obstacles at mucosal surfaces, such as poor mucus permeability and rapid mucociliary clearance, which hinder the effective delivery of mRNA beyond the nasal epithelium.^[^ ^5^ ^]^ In this study, we designed muco‐penetrating iLLNs with tunable p*K*_a_ values for enhanced intranasal mRNA delivery. By controlling the mixing ratios of ALC‐0315 to DOTMA, the p*K*_a_ of iLLNs was adjusted to the range of nasal mucosal pH (5.5–6.5), among which 5.94 was found to be an optimal p*K*_a_. In a transwell mucus diffusion model, iLLN‐2/mRNA complexes achieved the best muco‐penetrating performance among all tested formulations possibly by effectively avoiding entrapment within mucin fibers due to their superior muco‐inertness and nearly neutral PEGylated surfaces. Additionally, iLLN‐2/mRNA complexes were found to have superior endosomal escape and transfection ability in both A549 and DC2.4 cells over ALC‐LNP. Considering that ALC‐LNP has a similar p*K*_a_ value (6.09) to that of iLLN‐2,^[^ ^17^ ^]^ it can be reasoned that the membrane fusion activity of ALC‐0315 was strongly augmented when incorporated in combination with DOTMA.

The surface PEG density is another critical parameter that needs to be calibrated carefully when designing mucus‐permeable nanoparticles. Previous studies have shown that at least 5 wt% PEG coatings are required to effectively shield the particle surface from adhesive interactions with mucus.^[^ ^8^ ^]^ However, too high PEG coverage may limit cellular internalization and in vivo organ uptake of nanoparticles by providing a steric hindrance effect.^[^ ^38^, ^41^ ^]^ In this regard, we attempted to find an optimal PEG‐lipid density of iLLN‐2/mRNA complexes that would result in efficient mRNA delivery across airway mucosa. In vivo bioluminescence imaging revealed that raising DSPE‐PEG content from 0.5 to 5 mol% led to a drastic reduction in FLuc mRNA expression within the nasal cavity of BALB/c mice. These data are in agreement with the literature reporting a decline in in vivo transfection efficiency with increasing PEG‐lipid content up to ≈5 mol%.^[^ ^10^ ^]^ Based on this observation, we selected iLLN‐2/mRNA complexes containing 0.5 mol% DSPE‐PEG for the rest of studies. When nasally administered in mice, iLLN‐2/mRNA complexes outperformed ALC‐LNP and cLLN/mRNA complexes by providing up to ≈60‐fold and ≈8‐fold higher reporter gene expression in the nasal cavity. Ex vivo organ imaging revealed that iLLN‐2/mRNA complexes achieved highly localized FLuc expression in the nose and lung, demonstrating their capability to overcome airway mucus barrier.

Despite the relatively low PEG‐lipid content (0.5 mol%), iLLN‐2/mRNA complexes were found to have superior mucus stability over ALC‐LNP containing a higher PEG‐lipid content (1.6 mol%).^[^ ^1^ ^]^ In mucin‐saturated solution, ALC‐LNP showed a decrease in the derived count rate (a DLS parameter representative of the absolute scattering intensity) within 5 min, indicating its rapid disintegration. Upon contact with protein‐rich biofluids (e.g., blood, plasma), mRNA‐LNPs are known to dynamically exchange the lipid components with proteins, generating the so‐called “protein corona” which influences their biodistribution, efficacy, and toxicity in vivo.^[^ ^42^ ^]^ A recent lipid profiling study has elucidated that ALC‐LNP dissociates immediately after exposure to human plasma via desorption of lipid components, such as PEG‐lipid and ALC‐0315, resulting in a reduction in the particle molecular mass and DLS light scattering signal.^[^ ^43^ ^]^ Since the lipid displacement is thought to occur mainly via hydrophobic interactions,^[^ ^44^ ^]^ the poor mucus stability of ALC‐LNP was likely caused by its interactions with mucin proteins having abundant hydrophobic domains.^[^ ^7^ ^]^ It has been documented that the alkyl chain length of PEG‐lipids strongly impacts their desorption rates from LNPs. For instance, PEG‐lipids with longer (C18) chains had slower desorption from LNPs in circulation than those with shorter (C14) chains.^[^ ^44^, ^45^ ^]^ Hence, the existence of DSPE‐PEG (C18 PEG‐lipid), triolein (a triglyceride having three C18 oleoyl chains), and DOTMA (C18 cationic lipid) might, at least partly, render iLLN‐2/mRNA complexes more resistant to mucin‐mediated disintegration by increasing hydrophobic attractions among the lipid components.^[^ ^46^ ^]^

To date, only a few studies have explored the possibility of mRNA‐LNPs for intranasal administration,^[^ ^11^, ^47^ ^]^ but most of them have shown limited mucosal IgA response. In a previous mouse study, two 2‐µg doses of nasally administered ALC‐LNP did not induce mucosal IgA and systemic IgG production despite detectable levels of IgG response in NLF.^[^ ^47c^ ^]^ In addition, a prime‐boost nasal vaccination with 10 µg of mRNA/poly(amine‐*co*‐ester) complexes generated antigen‐specific IgG in BALF, but not IgA response, suggesting that IgA‐secreting B cells in lymph nodes were not effectively recruited to the respiratory tract.^[^ ^48^ ^]^ Encouragingly, a prime‐boost intranasal immunization with the same dose (10 µg) of iLLN‐2/mRNA complexes elicited SARS‐CoV‐2 spike‐specific mucosal IgA and IgG response in NLF as well as systemic IgG response in serum. This finding provides evidence that intranasal vaccination with iLLN‐2/mRNA complexes could not only transfect APCs to facilitate their antigen presentation, but also promote recruitment of IgA‐secreting B cells to the nasal passage.

A major obstacle to nasal mRNA vaccines is their tendency to cause pulmonary inflammation and mortality. Nasally administered ALC‐LNP at 10‐µg dose caused the death of 50% of the dosed mice within 4 days with a concomitant body weight loss, which is consistent with the literature documenting the lethal effect of mRNA‐LNPs at doses as low as 5 µg.^[^ ^40^ ^]^ On the contrary, iLLN‐2/mRNA complexes did not cause any noticeable weight loss for 28 days. Clinical chemistry analysis detected elevated levels of albumin, total protein, and globulin in the mice dosed with ALC‐LNP, reflecting its highly inflammatory nature.^[^ ^49^ ^]^ This finding was further supported by the increased serum TNF‐α levels and severe accumulation of neutrophils in the lungs. In contrast, the same nasal dose (10 µg) of iLLN‐2/mRNA complexes was well tolerated with negligible levels of local and systemic inflammation. Previously, the inflammatory property of mRNA‐LNPs has been thought to originate mainly from their ionizable lipid components.^[^ ^50^ ^]^ Ndeupen et al. reported that the removal of ionizable lipids from mRNA‐LNPs greatly mitigated their immunostimulatory effects.^[^ ^40a^ ^]^ However, this is not the case in our study because there was not much difference in the administered amount of ALC‐0315 between ALC‐LNP (135.4 µg/mouse) and iLLN‐2/mRNA complexes (104.8 µg/mouse; Table S2, Supporting Information). Interestingly, multiple studies have found anti‐inflammatory activities of triolein and *β*‐sitosterol, the components existing only in iLLN‐2/mRNA formulation.^[^ ^51^ ^]^ In a murine model of lung chronic infection, *β*‐sitosterol treatment effectively alleviated pulmonary inflammation by lowering proinflammatory cytokines involved in neutrophil chemotaxis.^[^ ^52^ ^]^ In this perspective, we speculate that the anti‐inflammatory effects of triolein and *β*‐sitosterol would have probably contributed to the superior tolerability of nasally administered iLLN‐2/mRNA complexes. Collectively, the present study demonstrates the potential of iLLN‐2/mRNA complexes as safer and more efficacious intranasal mRNA vehicles.

## 4 Experimental Section

### Materials

ALC‐0315, ALC‐0159, 1,2‐di‐*O*‐octadecenyl‐3‐trimethylammonium propane (DOTMA), 1,2‐dioleoyl‐*sn*‐glycero‐3‐phosphoethanolamine (DOPE), 1,2‐distearoyl‐*sn*‐glycero‐3‐phosphorylcholine (DSPC), 1,2‐distearoyl‐*sn*‐glycero‐3‐phosphoethanolamine‐polyethylene glycol 2000 (DSPE‐PEG) and triolein were purchased from MedChemExpress (Monmouth Junction, NJ, USA). Cholesterol, GelRed nucleic acid staining dye, porcine stomach type III mucin, and Triton X‐100 were obtained from Sigma‐Aldrich (St. Loius, MN, USA). *β*‐Sitosterol was a product of Abcam (Cambridge, UK). 6‐(*p*‐Toluidino)−2‐naphthalenesulfonic acid sodium salt (TNS) was purchased from Santa Cruz Biotechnology (Dallas, TA, USA). ONE‐Glo Luciferase Assay reagent, VivoGlo luciferin, and nuclease‐free water were obtained from Promega Corporation (Madison, WI, USA). 5‐Methoxyuridine‐modified firefly luciferase mRNA (FLuc mRNA) and SARS‐CoV‐2 Delta variant (B.1.617.2) spike protein‐encoding mRNA (PVX1010 mRNA)^[^ ^32^ ^]^ were produced using a proprietary custom process at TriLink BioTechnologies (San Diego, CA, USA). Cy5‐tagged FLuc mRNA (Cy5‐mRNA) was bought from ApexBio Technology (Houston, TX, USA). AlamarBlue cell viability assay reagent, ACK lysing buffer, DiI (1,1′‐dioctadecyl‐3,3,3′,3′‐tetramethylindocarbocyanine perchlorate), Hoechst 33 342, Lab‐Tek II chambered coverglass, LysoTracker Green DND‐26, Pierce detergent‐compatible Bradford assay kit and Quant‐iT RiboGreen RNA assay kit were bought from Thermo Fisher Scientific (Waltham, MA, USA). SARS‐CoV‐2 spike protein ELISA kit (GeneTex, USA), OptEIA mouse TNF ELISA kit (BD Biosciences, USA), and mouse interferon‐γ (IFN‐γ) single‐color ELISPOT kit (Cellular Technology Ltd, USA) were used as per manufacturer instructions. All other chemicals and reagents were of analytical grade.

### Preparation of iLLN and cLLN Formulations

An emulsification/solvent evaporation technique was used to produce iLLN and cLLN formulations, Briefly, ALC‐0315, DOTMA, DOPE, *β*‐sitosterol, triolein, and DSPE‐PEG were co‐dissolved at specified weight ratios (**Table** **1**) in 1 mL of chloroform/ethanol mixture (4:1, v/v) in a 15‐mL conical tube. The total lipid concentration was set to 10 mg mL^−1^. After 5 mL of nuclease‐free water was added, the mixture was vortexed for 10 s and then sonicated for 2 min using a HTU Soni‐130 ultrasonic homogenizer (20 kHz, 130 Watt). The oil‐in‐water emulsion was transferred to a 100‐mL round‐bottom flask and the solvents were evaporated using a Hei‐VAP rotary evaporator (Heidolph, Germany) for 10 min at 60 °C. The resultant iLLN and cLLN formulations were stored at 4 °C until use.

### Formation of iLLN/mRNA and cLLN/mRNA Complexes

iLLN in 5 µL of nuclease‐free water was mixed with 100 ng of mRNA in 5 µL of nuclease‐free water at the nitrogen‐to‐phosphate (N/P) ratio of 6. The volume ratio of iLLN suspension and mRNA solution was set to 1:1. The mixture was incubated for 10 min at 25 °C to form iLLN/mRNA complexes. For comparison, cLLN/mRNA complexes were produced in the same manner.

### Preparation of ALC‐LNP

mRNA was encapsulated in ALC‐LNP using the same lipid composition as that of BNT162b2 vaccine.^[^ ^1^ ^]^ Briefly, mRNA was prepared in 10 mm sodium acetate buffer (pH 4.8) to obtain the aqueous phase. Lipids were solubilized in ethanol at a molar ratio of 46.3:1.6:9.4:42.7 (ALC‐0315:ALC‐0159:DSPC:cholesterol) to form the organic phase. The aqueous and organic phases (N/P ratio = 6) were mixed at a volume ratio of 3:1 and a flow rate of 12 mL min^−1^ using the NanoAssemblr Ignite microfluidics platform (Precision Nanosystems, Canada). The resultant ALC‐LNP was buffer‐exchanged with normal saline and concentrated using a Vivaspin 20 centrifugal filter (*M*_w_ cut‐off = 30 kDa). The amount of encapsulated mRNA was quantified using the Quant‐iT RiboGreen RNA assay kit, as described in the previous report.^[^ ^43^ ^]^

### Nanoparticle Characterization

Z‐average size, polydispersity index, and zeta potential of bare iLLN and cLLN formulations were examined in nuclease‐free water by dynamic light scattering (DLS) using a Zetasizer Ultra Red (Malvern Panalytical, UK). DLS measurements of iLLN/mRNA and cLLN/mRNA complexes formulated with PVX1010 mRNA were conducted in pH 6 saline. Each sample was diluted fivefold with pH 6 saline and measured at 25 °C in triplicate. For gel electrophoresis, iLLN/mRNA and cLLN/mRNA complexes formulated with PVX1010 mRNA were mixed with an equal volume of pH 6 saline and incubated for 1 h at 25 °C. The samples were then run on a 1% agarose gel containing GelRed dye in Tris‐acetate‐EDTA buffer at 100 V for 40 min. A RiboRuler high‐range RNA ladder (Thermo Fisher Scientific, USA) was used for size comparison. The gel image was captured with an iBright FL1500 Imaging System (Invitrogen, USA). For cryo‐TEM imaging, Quantifoil grids were glow‐discharged for 1 min and then loaded with samples using a Vitrobot cryo‐plunger. The grids were observed under a Tecnai Arctica 200 kV electron microscope (FEI, USA) at the NTU Institute of Structural Biology. The apparent p*K*_a_ values of iLLNs were measured by TNS binding assay, as reported previously.^[^ ^28^ ^]^

### Evaluation of FLuc mRNA Translation Efficiency and Cell Viability

The human alveolar basal epithelial cell line A549 and mouse dendritic cell line DC2.4 (ATCC, USA) were maintained in 10% fetal bovine serum‐supplemented DMEM and RPMI 1640 media, respectively. The cells were seeded in a white‐walled 96‐well plate at a density of 10^4^ cells well^−1^ and cultured for 24 h. Each well was then treated with iLLN/mRNA or cLLN/mRNA complexes formulated with 100 ng of FLuc mRNA at a N/P ratio of 6. For comparison, other wells were treated with ALC‐LNP at the same dose of FLuc mRNA (100 ng well^−1^). After 48 h, the cells were rinsed with 100 µL of PBS before the addition of ONE‐Glo Luciferase Assay reagent (100 µL well^−1^). After 5 min, the relative luminescence unit (RLU) of the cell lysate was measured on a Spark 10 M microplate reader (Tecan Group, Switzerland). The results were standardized for protein content using the detergent‐compatible Bradford assay kit and expressed as RLU/mg protein. To assess the cell viability, a separate group of cells was seeded in a black‐walled 96‐well plate (10^4^ cells well^−1^) and treated for 48 h with iLLN/mRNA or cLLN/mRNA complexes, as described above. After rinsing with PBS, 100 µL of AlamarBlue reagent (10% in the culture media) was added to each well and incubated for 2 h. Fluorescence intensity (FI) was measured using the Spark 10 M microplate reader with an excitation wavelength of 560 nm and an emission wavelength of 590 nm. Cell viability was determined as a percentage of FI of analyzed cells relative to untreated controls.

### Evaluation of PVX1010 mRNA Transfection Efficacy and Cell Viability

A549 cells were seeded in a 12‐well plate at a density of 10^5^ cells well^−1^ and cultured for 24 h. Each well was then treated with iLLN/mRNA or cLLN/mRNA complexes formulated with 200 ng of PVX1010 mRNA (N/P ratio = 6). For comparison, other wells were treated with ALC‐LNP at the same dose of PVX1010 mRNA. After 48 h, the supernatant was withdrawn and centrifuged for 10 min at 860 × *g* at 4 °C. The spike S1 concentration in the supernatant was examined using a SARS‐CoV‐2 spike protein ELISA kit (GeneTex, USA). To check viability, 1 mL of AlamarBlue assay reagent (10% in the culture media) was added into each well of the plates. After 2 h at 37 °C, 200 µL of the media were placed in black‐bottomed 96‐well plates, and FI (excitation at 560 nm; emission at 590 nm) was measured on the Spark 10 M microplate reader. Cell viability was determined as a percentage of FI of analyzed cells relative to untreated controls.

### Confocal Laser Scanning Microscopy

A549 cells were seeded on an 8‐well Lab‐Tek II chambered coverglass at a density of 2 × 10^4^ cells well^−1^ and cultured for 24 h. Each well was then treated with ALC‐LNP or iLLN‐2/mRNA complexes formulated with 200 ng of Cy5‐mRNA at an N/P ratio of 6. After 4 h, the cells were rinsed with 100 µL of PBS, followed by staining with LysoTracker Green DND‐26 (200 nM) for 1 h and Hoechst 33 342 (10 µg mL^−1^) for 20 min. After rinsing with PBS, the cells were fixed with 4% paraformaldehyde for 15 min and observed under a LSM980 laser‐scanning confocal microscope (Zeiss, Germany) equipped with a 63× oil‐immersion objective lens. The fluorescent signal of Cy5‐mRNA in each cell area was measured using the RGB Measure plugin in ImageJ 1.54g software (National Institutes of Health, USA). The endosomal escape efficiency was determined by the ratio of Cy5 signal in the merged channel (mRNA escaped from endosomes) to the total fluorescent signal in the Cy5‐mRNA channel (internalized mRNA).^[^ ^53^ ^]^

### Mucus Penetration Study

Fluorescently labeled iLLN, cLLN, and ALC‐LNP were formulated by adding DiI dye (0.2 mol% of total lipid) into the lipid mixture, according to a previous report.^[^ ^54^ ^]^ DiI‐labeled iLLN‐2 lacking triolein was prepared using the same lipid composition as that of DiI‐labeled iLLN‐2, except for the absence of triolein. A transwell mucus diffusion model was prepared by adding 25 µL of 5% mucin in PBS (pH 6) into an 8.0‐µm pore polycarbonate membrane of 6.5‐mm Transwell inserts (Corning cat#3422). After the receptor chamber was filled with 600 µL of PBS (pH 6), the plate was incubated at 37 °C with shaking at 100 rpm for 15 min to remove air bubbles from the mucin layer.^[^ ^55^ ^]^ Next, 100 µL of DiI‐labeled iLLNs, cLLN or their corresponding mRNA complexes (1 µg of PVX1010 mRNA/chamber, N/P ratio = 6) were loaded onto the top of the mucin layer and then incubated with shaking at 100 rpm. For comparison, other mucin layers were treated with ALC‐LNP at the same dose of PVX1010 mRNA (1 µg/chamber). At predetermined time intervals, samples (100 µL) were withdrawn from the receptor chamber and kept at 4 °C. At each time interval, 100 µL of fresh PBS (pH 6) was replenished in the receptor chamber to keep the volume constant. The fluorescence intensity was recorded in a black‐bottomed 96‐well plate using a Spark 10 M microplate reader (Tecan Group, Switzerland) with an excitation wavelength at 525 nm and an emission wavelength at 565 nm. For each formulation, positive control experiments were performed under the same condition without the mucin layer. Mucus penetration was determined as a percentage of the fluorescence intensity relative to the positive control. Cumulative corrections were made for the previously withdrawn samples. The apparent permeability coefficient (*P*_app_) was calculated using the following equation:^[^ ^56^ ^]^

(1) $$P_{\mathrm{app}}=\frac{dQ}{dt}\times \frac{1}{A\times C_{0}}$$

where d*Q*/d*t* is the rate of DiI appearance in the receptor chamber, *A* is the surface area of the transwell (0.33 cm^2^) and *C*_0_ is the initial DiI concentration in the donor chamber.

### Mucus Stability Tests

The stability of iLLN‐2/mRNA and cLLN/mRNA complexes in mucin‐saturated solution was examined according to the previous report with some modifications.^[^ ^57^ ^]^ Briefly, a saturated mucin solution was prepared by dispersing 0.08% (w/v) mucin in de‐ionized water with overnight stirring. After centrifugation at 6000 × *g* for 20 min at 4 °C, the mucin‐containing supernatant was collected. Then, 20 µL of iLLN‐2/mRNA or cLLN/mRNA complexes (200 ng of PVX1010 mRNA, N/P ratio = 6) were mixed with 780 µL of the mucin solution and then incubated at 37 °C on a shaking board set at 100 rpm. The stability of the complexes following mucin exposure was examined by monitoring the derived count rate over 180 min using a Zetasizer Ultra Red (Malvern Panalytical, UK).

### In Vivo and Ex Vivo Bioluminescence Imaging

All animal procedures were performed in accordance with the approved protocol 221 681 from the Institutional Animal Care and Use Committee (IACUC) at the Biological Resource Centre of A*STAR, Singapore. Female BALB/c mice (5–6 weeks old) were acquired from InVivos Pte Ltd (Singapore) and randomly allocated to different experimental groups: iLLN/mRNA complexes (*n* = 4), cLLN/mRNA complexes (*n* = 4), ALC‐LNP (*n* = 4) and mock control (*n* = 2). After anesthesia with ketamine (75 mg kg^−1^)/xylazine (5 mg kg^−1^), the mice were held by hand in an upright position and intranasally administered with a total 60 µL of an isotonic glucose solution (5% w/v) containing the prepared formulations (10 µg of FLuc mRNA/mouse). Once every 10 µL was given dropwise into one nostril using a 10‐µL pipette, the footpad was pinched to make the mouse take a deep breath, allowing the instilled solution to reach the lower respiratory tract.^[^ ^58^ ^]^ For screening of optimal DSPE‐PEG contents, separate groups of mice were intranasally administered with a 2‐µg mRNA dose of iLLN‐2/mRNA complexes with varying DSPE‐PEG contents (0.5, 1, 3, and 5 mol% of total lipid). After 4 h, each mouse was re‐anesthetized and subjected to intranasal (50 µL) and intraperitoneal (150 µL) administration of VivoGlo luciferin (15 mg mL^−1^ in PBS).^[^ ^59^ ^]^ After stabilization for 10 min, the whole‐body luminescence signal was acquired on the IVIS Spectrum imaging system (PerkinElmer, USA). Organs (nose, lung, spleen, liver, kidney, heart) were excised immediately and immersed in 1 mL of VivoGlo luciferin (0.3 mg mL^−1^ in PBS) before ex vivo luminescence imaging. The total flux in each organ was quantified using the Living Image software (PerkinElmer, USA).

### Intranasal Vaccination Study

Female BALB/c mice (5–6 weeks old, InVivos Pte Ltd) were intranasally immunized with iLLN/mRNA complexes (*n* = 5) or ALC‐LNP (*n* = 8) at the same dose (10 µg of PVX1010 mRNA/mouse) on day 0 (prime) and day 21 (boost), using the administration procedure described above. Unimmunized mice matched for age and gender (*n* = 3) were used as a negative control. The body weight and survival time were monitored throughout the experiment. Blood was collected on days 7, 14, 21, and 28 via submandibular bleeding in Microvette clotting activator tubes (Sarstedt, Germany). After coagulation, serum was separated by centrifugation at 10 000 × *g* for 5 min and then stored at −20 °C. On day 28 post‐prime vaccination, the mice were euthanized via CO_2_ inhalation, and nasal lavage fluid (NLF) was collected using a previously described method.^[^ ^60^ ^]^ Briefly, the jaws and tongues of the mice were removed to expose the nasopharynx opening. After flushing 100 µL of PBS containing 50 µm EDTA into the nasal cavity with a blunt needle, NLF was harvested at the nose opening. To collect bronchoalveolar lavage fluid (BALF), the lungs and trachea were exposed and a blunt‐tip needle was inserted into the trachea. After inflation of the lungs with 1000 µL of PBS, BALF was recovered by gentle manual suction.

### Clinical Chemistry, TNF‐α ELISA, and Histology

The collected sera (200 µL per mouse) were examined by a RX daytona+ clinical chemistry analyzer (Randox Laboratories, UK). Sera harvested from five unimmunized mice were used as a negative control. The serum levels of mouse TNF‐α were measured using OptEIA mouse TNF ELISA kit (BD Biosciences, USA). Histology was performed at the Advanced Molecular Pathology Laboratory (AMPL), A*STAR. Briefly, the nose and lung tissue were fixed with 10% neutral buffered formalin, paraffin‐embedded, and then sectioned at 3 µm thickness for hematoxylin and eosin (H&E) staining. The sections were immunostained with a rat anti‐mouse Ly6G monoclonal antibody (HyCult Biotech #HM1039, 1:50) and a goat anti‐rat IgG‐HRP conjugate (Abcam #ab97057, 1:100). The histological images were taken under an IX83 inverted microscope (Olympus, Japan) and analyzed by ImageJ 1.54g software (National Institutes of Health, USA).

### ELISPOT Assay

The mouse lungs were gently mashed through a 70‐µm cell strainer, treated with ACK lysing buffer, and then resuspended in RPMI 1640 media containing 10% heat‐inactivated fetal calf serum. After cell counting with a hemocytometer, 2 × 10^5^ cells were seeded into each well of a mouse IFN‐γ single‐color ELISPOT plate (Cellular Technology Ltd, USA) and stimulated for 24 h with a PepTivator SARS‐CoV‐2 Prot_S B.1.617.2 mutation pool (Miltenyi Biotec, Germany). The developed spots were counted using an IRIS ELISpot reader (Mabtech, Sweden) equipped with Mabtech Apex 1.1.9 software. The results were expressed as spot‐forming units (SFU) per million cells.

### Spike‐Specific IgA and IgG Measurements

MaxiSorp 96‐well flat‐bottom plates (Nunc, Denmark) were coated with 50 ng well^−1^ of recombinant SARS‐CoV‐2 B.1.617.2 spike protein (R&D System, USA) in carbonate‐bicarbonate buffer (pH 9.6) and incubated overnight at 4 °C. After aspiration, the wells were blocked with PBS containing 0.05% Tween‐20 and 3% non‐fat milk (PBST‐milk) for 1 h at room temperature. Mouse sera diluted 1:400 in PBST‐milk or undiluted nasal wash and BALF was transferred to the blocked wells and then incubated for 2 h at 37 °C. Plates were then washed three times with PBST and incubated with horseradish peroxidase‐conjugated goat anti‐mouse IgA (Invitrogen cat#62‐6720, 1:2500) and IgG (Promega cat#W4021, 1:5000). After washing three times with PBST, plates were developed with BioFX one‐component 3,3′,5,5′‐tetramethylbenzidine (TMB) substrate (Surmodics, USA) and then halted by 1 m HCl. Absorbance was subsequently measured at 450 nm on an Infinite 200 PRO microplate reader (Tecan Group, Switzerland).

### Anti‐PEG IgM Measurements

The generation of anti‐PEG IgM was examined by a previously reported method.^[^ ^61^ ^]^ Briefly, 50 µL of DSPE‐PEG in ethanol (200 nmol mL^−1^) was added to each well of PolySorp 96‐well flat‐bottom plates (Nunc, Denmark). After drying overnight, the wells were blocked with PBS containing 4% blot‐qualified BSA (Promega W3841) for 2 h at 37 °C. Mouse sera were serially diluted threefold from 1:100 up to 1:218 700 and incubated on the blocked wells for 1 h at 25 °C. Plates were washed five times with PBS and incubated for 1 h with horseradish peroxidase‐conjugated goat anti‐mouse IgM (Bethyl Laboratories A90‐101P, 1:20 000). After washing five times with PBS, plates were developed with SureBlue one‐component TMB substrate (SeraCare, USA) and then halted by TMB Stop solution (SeraCare, USA). Optical densities (ODs) were measured at 450 nm on a Spark 10 M microplate reader (Tecan Group, Switzerland). Endpoint titers were calculated as the dilution that showed an OD exceeding a 3× background. Samples with OD values below the limit of detection are assigned an arbitrary value of 50.^[^ ^62^ ^]^

### Fusion Activity Test

The membrane‐destabilizing activity was assessed according to the literature with slight modifications.^[^ ^34^ ^]^ Blood was freshly collected from BALB/c mice via submandibular bleeding and rinsed thrice with normal saline by repeated centrifugation at 1000 × *g* for 5 min at 4 °C. Then, 250 µL of erythrocyte suspension (2 × 10^8^ cells mL^−1^) was mixed with 250 µL of iLLN formulations diluted in PBS (pH 5.5, 6.5, or 7.4). The final total lipid concentration was fixed at 30 µm. The equal volume of 1% Triton X‐100 solution and normal saline served as a positive and negative control, respectively. After incubation for 30 min at 37 °C, the mixture was centrifuged at 1000 × *g* for 5 min at 4 °C and 100 µL of the supernatant was transferred to a transparent 96‐well plate. The absorbance of the leaked hemoglobin at 545 nm was measured using a Spark 10 M microplate reader (Tecan Group, Switzerland). The fusion activity was calculated using the following equation:

(2) $$\mathrm{Fusion}\;\mathrm{activity}\;\;\left(\%\right)=\;\frac{\left[A_{\mathrm{sample}}-A_{\mathrm{NC}}\right]}{\left[A_{\mathrm{PC}}-A_{\mathrm{NC}}\right]}\times 100$$

where *A*_PC_, *A*_NC_, and *A*_sample_ represent the absorbance obtained with the positive control, negative control, and tested sample, respectively.

### Statistical Analysis

All data are presented as mean ± standard deviation (SD). Statistical analysis was performed using a two‐tailed unpaired Student's *t*‐test for two groups or an ordinary one‐way ANOVA with Tukey's post hoc test for three or more groups, employing GraphPad Prism 9.4.1 (GraphPad Software, USA). Statistical differences in the mouse survival rates were analyzed by a log‐rank test. Significance was determined at *p*‐values smaller than 0.05.

## Conflict of Interest

The authors declare no conflict of interest.

## Author Contributions

N.M. and K.H.B. contributed equally to this work. Y.Y.Y. and K.H.B. were involved in the conception and design of the study. N.M., K.H.B., J.L., and M.J.Y.A. conducted nanoparticle synthesis and in vitro characterization experiments. N.M., K.H.B., J.L., Z.W.C., and Y.J.T. performed animal experiments and downstream analysis. N.M., K.H.B., Z.W.C., and M.J.Y.A. performed data analysis and drafted the manuscript. J.L. created the organ diagram in Figure 5. L.F.P.N., L.R., K.P.W., and Y.Y.Y. provided guidance and edited the manuscript. Y.Y.Y. supervised the project. All authors reviewed and approved the final manuscript.

## Supporting information



Supporting Information

## Data Availability

The data that support the findings of this study are available from the corresponding author upon reasonable request.
