# Supplementary material for: Muco‐Penetrating Lipid Nanoparticles Having a Liquid Core for Enhanced Intranasal mRNA Delivery
Source: Adv Sci (Weinh). 2025 Jan 30;12(11):2407383. doi: 10.1002/advs.202407383 (PMC11923898; doi:10.1002/advs.202407383)
Supplement: Supplementary file 1 — Supporting Information [file ADVS-12-2407383-s001.docx]

Supporting Information

Muco-Penetrating Lipid Nanoparticles Having a Liquid Core for Enhanced Intranasal mRNA Delivery

Nipuni Maniyamgama, Ki Hyun Bae, Zi Wei Chang, Jialing Lee, Melgious J. Y. Ang, Yong Jie Tan, Lisa F. P. Ng, Laurent Renia, Kevin P. White and Yi Yan Yang*

**Figure S1.** Polydispersity index of bare iLLN and cLLN formulations in nuclease-free water. Mean ± SD (*n* = 3).


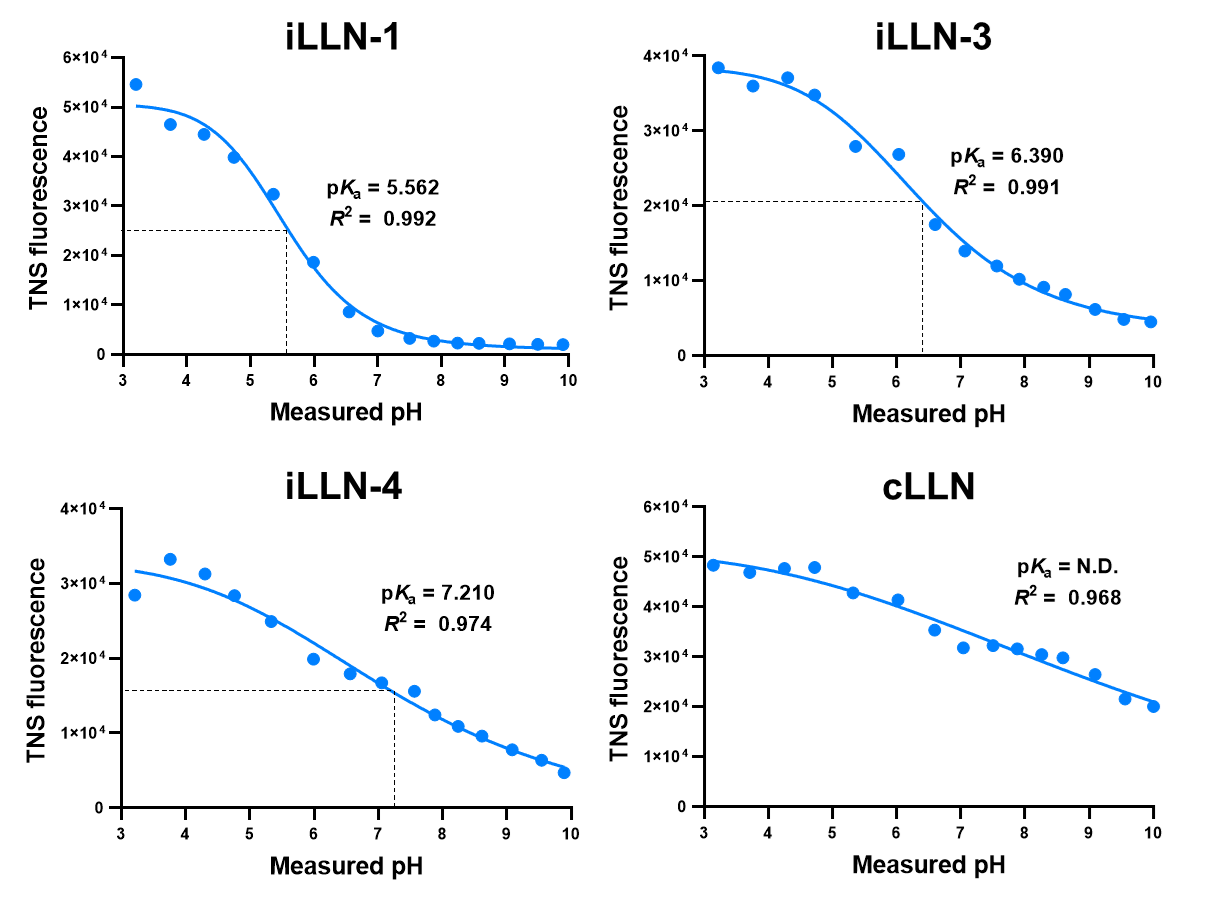


**Figure S2.** Representative TNS fluorescence titration curves of iLLN-1, iLLN-3, iLLN-4 and cLLN.


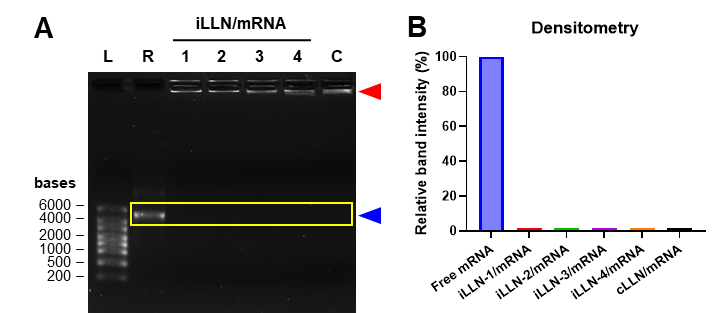


**Figure S3.** (A) Gel electrophoresis analysis of iLLN/mRNA and cLLN/mRNA complexes formulated with PVX1010 mRNA. L: ladder, R: free mRNA, 1-4: iLLN-1/mRNA to iLLN-4/mRNA complexes, C: cLLN/mRNA complex. The blue and red arrowheads indicate the location of free mRNA and its corresponding complexes, respectively. The yellow box indicates the area selected for densitometry analysis. (B) Relative band intensity of free mRNA and its complexes with iLLNs and cLLN was determined by densitometry analysis using ImageJ software.


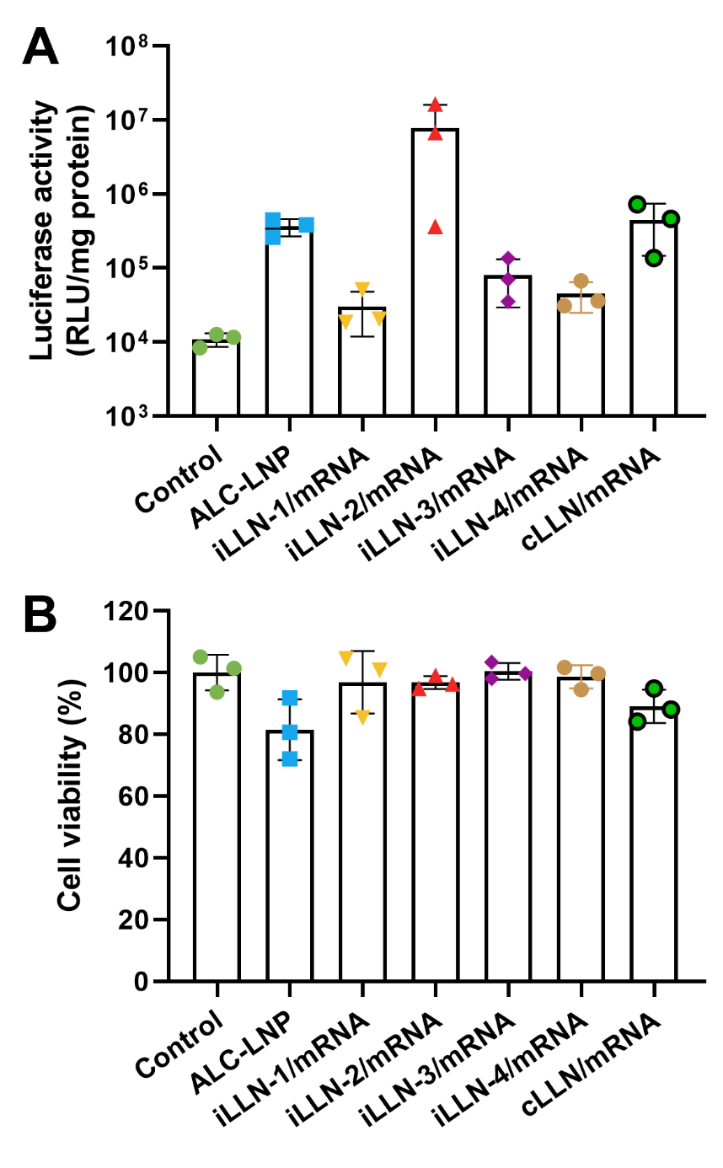


**Figure S4.** (A) Luciferase expression level and (B) viability of DC2.4 cells treated for 48 h with ALC-LNP, iLLN/mRNA or cLLN/mRNA complexes formulated with FLuc mRNA. Mean ± SD (*n* = 3).


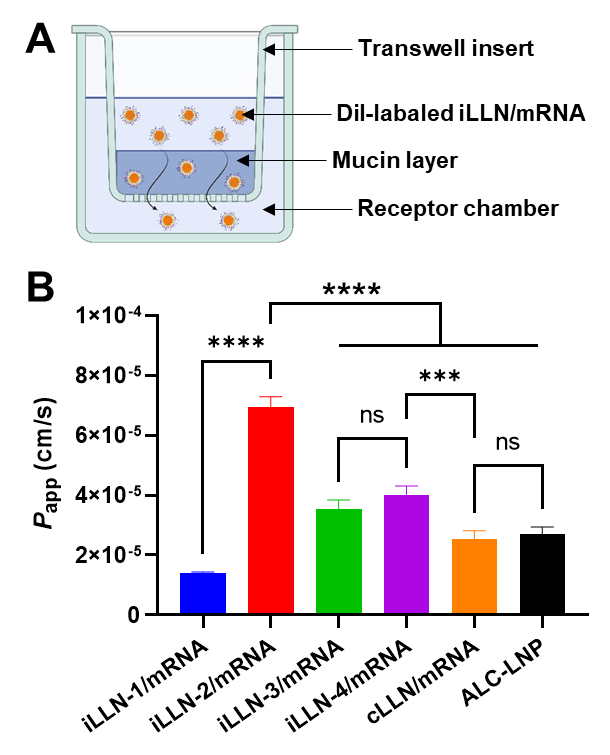


**Figure S5.** (A) Schematic illustration of the transwell mucus diffusion model used for the mucus penetration study. (B) Apparent permeability coefficient (*P*_app_) values of ALC-LNP, iLLN/mRNA or cLLN/mRNA complexes. Mean ± SD (*n* = 3); ^****^*P* < 0.0001; ^***^*P* < 0.001; ns: nonsignificant.


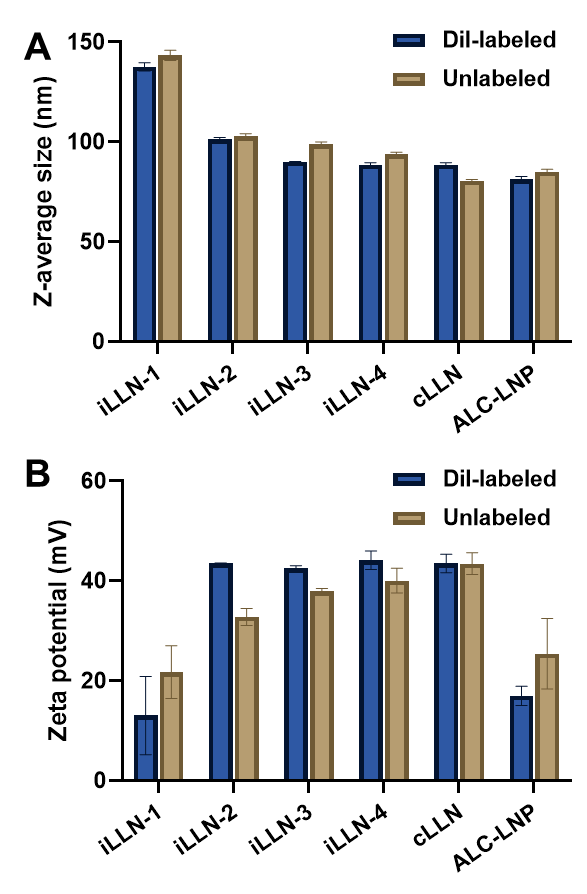


**Figure S6.** (A) Z-average size and (B) zeta potential of DiI-labeled or unlabeled iLLN, cLLN and ALC formulations in nuclease-free water. Mean ± SD (*n* = 3).


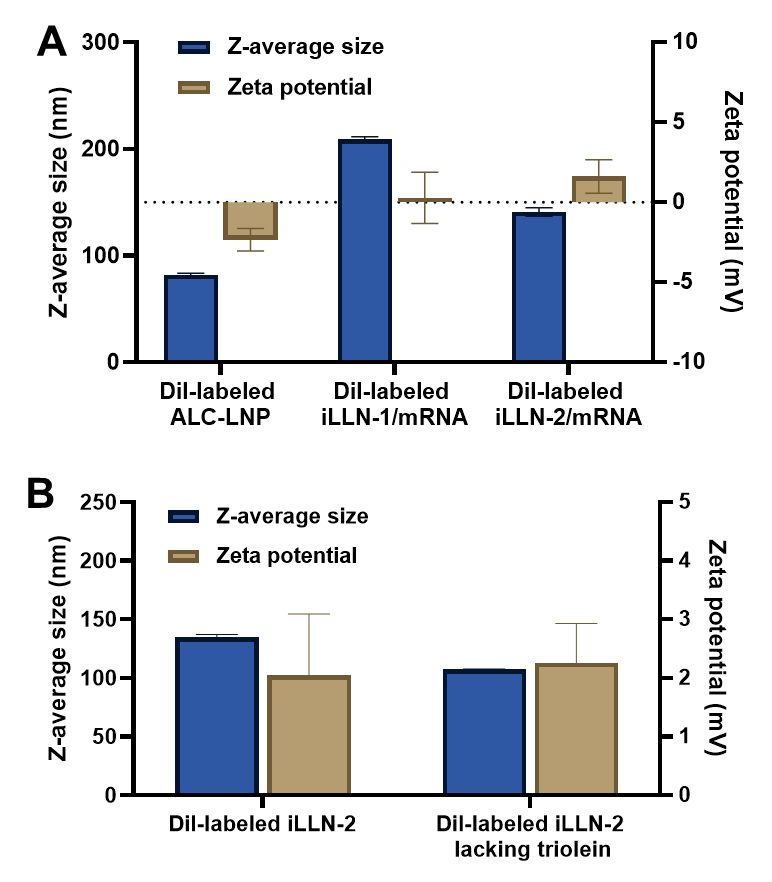


**Figure S7.** Z-average size and zeta potential of (A) DiI-labeled ALC-LNP, iLLN-1/mRNA, iLLN-2/mRNA, (B) DiI-labeled iLLN-2 and their counterpart lacking triolein, measured in PBS (pH 6). Mean ± SD (*n* = 3).


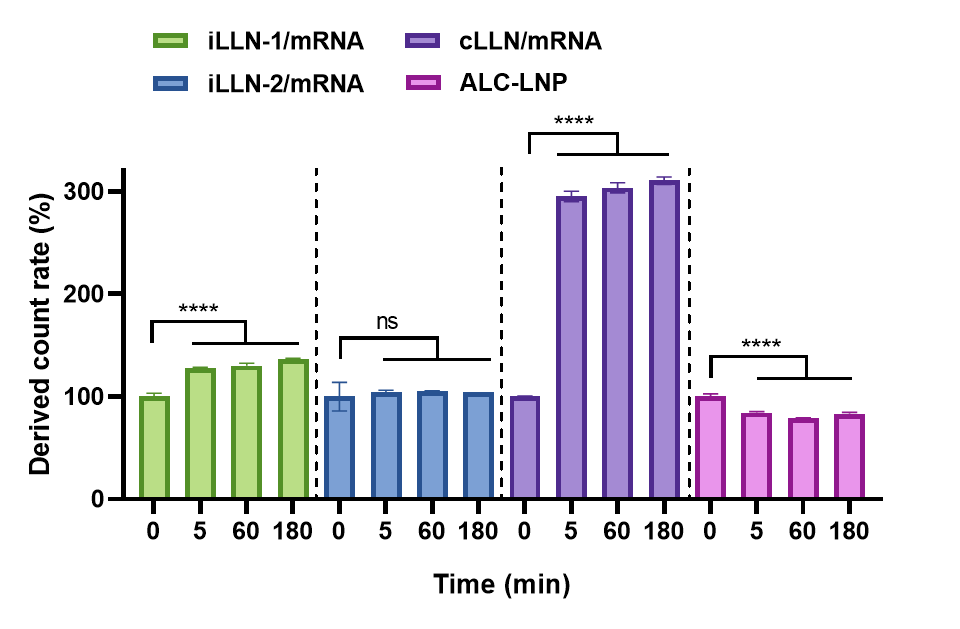


**Figure** **S8.** Time-course variations in the derived count rate of ALC-LNP, iLLN-1/mRNA, iLLN-2/mRNA and cLLN/mRNA complexes in mucin-saturated solution. Mean ± SD (*n* = 3); ^****^*P* < 0.0001; ns: nonsignificant.


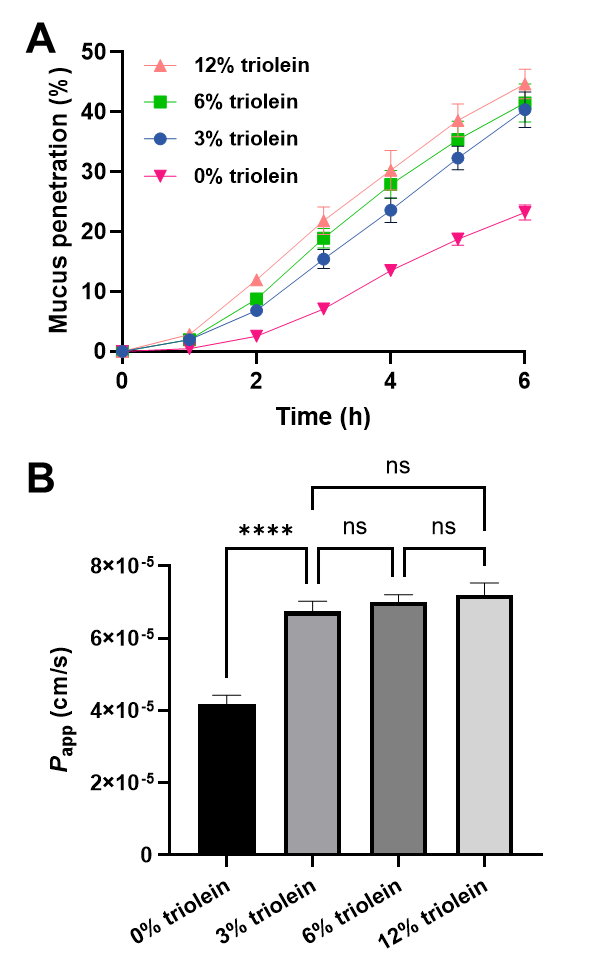


**Figure S9.** Effect of triolein fraction on (A) mucus permeability and (B) *P*_app_ values of iLLN-2 formulations. Mean ± SD (*n* = 3); ^****^*P* < 0.0001; ns: nonsignificant (one-way ANOVA with Tukey's post hoc test).


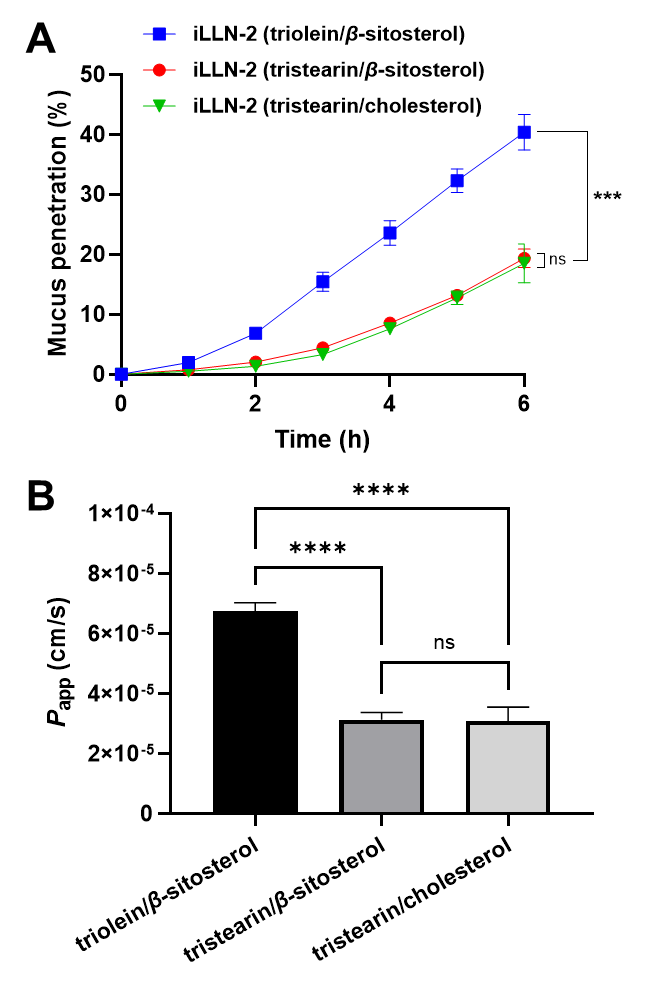


**Figure S10.** Effect of triolein and *β*-sitosterol substitution on (A) mucus permeability and (B) *P*_app_ values of iLLN-2 formulations. Mean ± SD (*n* = 3); ^****^*P* < 0.0001; ^***^*P* < 0.001; ns: nonsignificant (one-way ANOVA with Tukey's post hoc test).


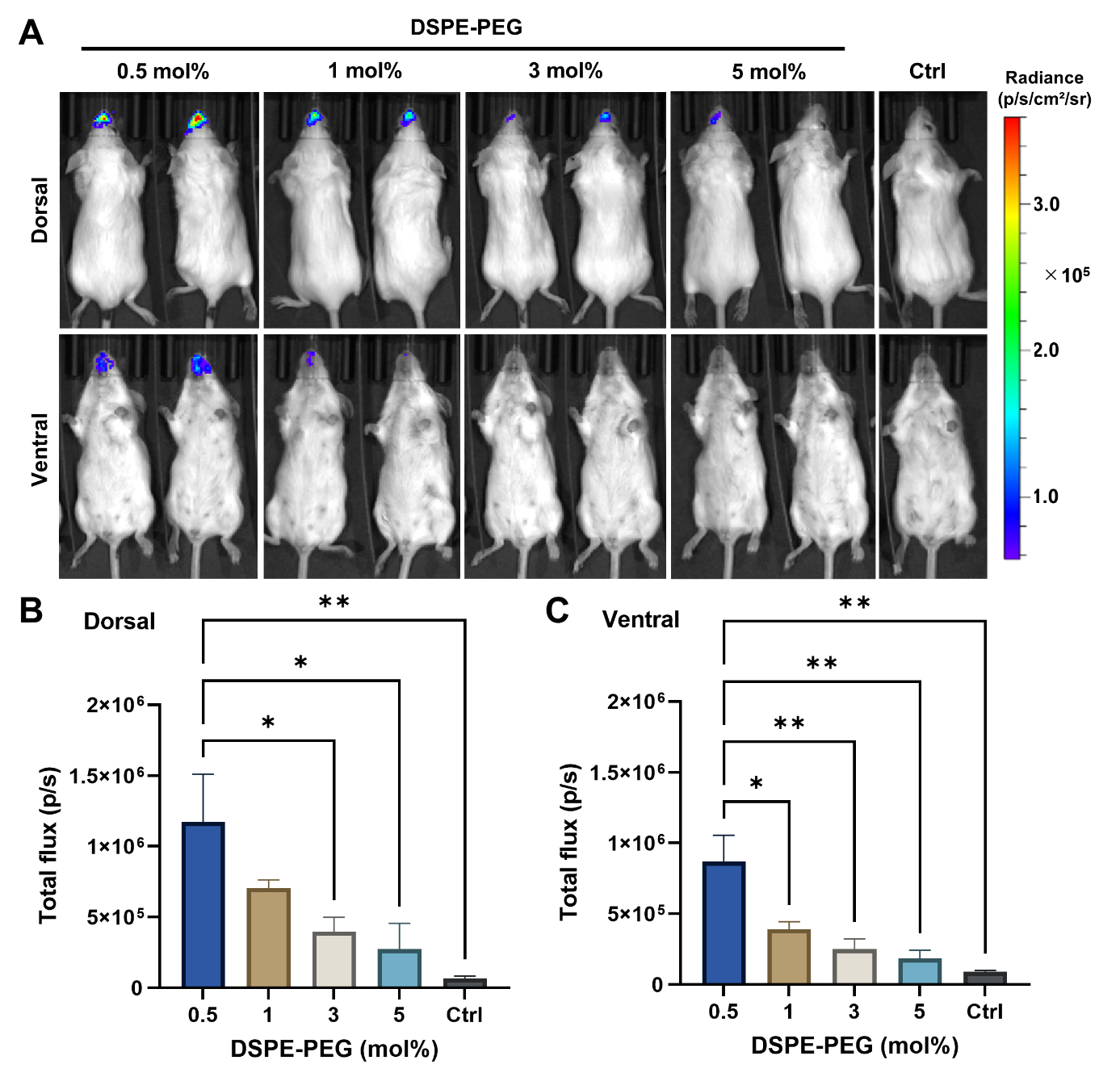


**Figure S11.** (A) Representative bioluminescence images of BALB/c mice at 4 h after intranasal administration with a 2-µg dose of iLLN-2/mRNA complexes with varying DSPE-PEG contents (0.5, 1, 3 and 5 mol% of total lipid). Top and bottom panels show the whole-body images on dorsal and ventral sides, respectively. Total flux values of the nasal cavity measured on (B) dorsal and (C) ventral sides. Mean ± SD (*n* = 2); ^**^*P* < 0.01; ^*^*P* < 0.05.


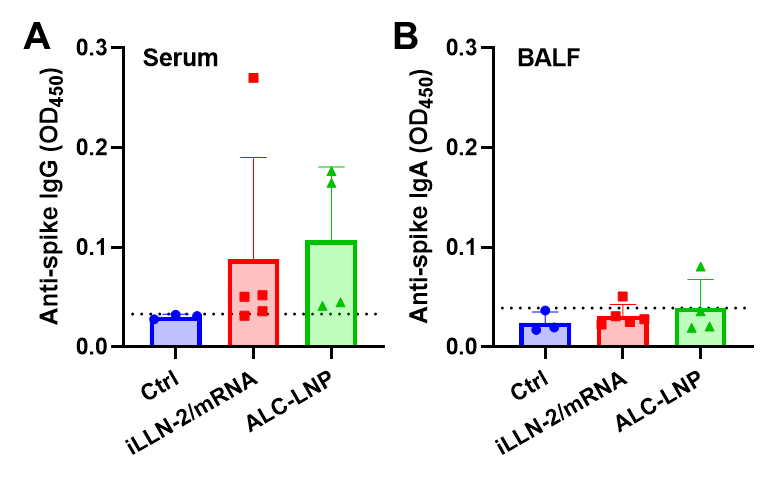


**Figure S12.** Levels of (A) anti-spike IgG in serum and (B) anti-spike IgA in BALF on day 28 following intranasal immunization with ALC-LNP or iLLN-2/mRNA complexes (10 µg mRNA per mouse). The dotted line indicates the limit of detection. Mean ± SD (*n* = 3 for Ctrl, *n* = 5 for iLLN-2/mRNA, *n* = 4 for ALC-LNP).

**Figure S13.** Anti-PEG IgM titers from mouse sera collected on day 7, 14 and 21 following intranasal immunization with ALC-LNP or iLLN-2/mRNA complexes (10 µg mRNA per mouse). The dotted line indicates the limit of detection. Mean ± SD (*n* = 5 for iLLN-2/mRNA, *n* = 4 for ALC-LNP); ns: nonsignificant.

**Figure S14.** Relative body weight changes of the mice dosed with ALC-LNP or iLLN-2/mRNA complexes (10 µg mRNA per mouse). Mean ± SD (*n* = 5 for iLLN-2/mRNA, *n* = 4 for ALC-LNP).


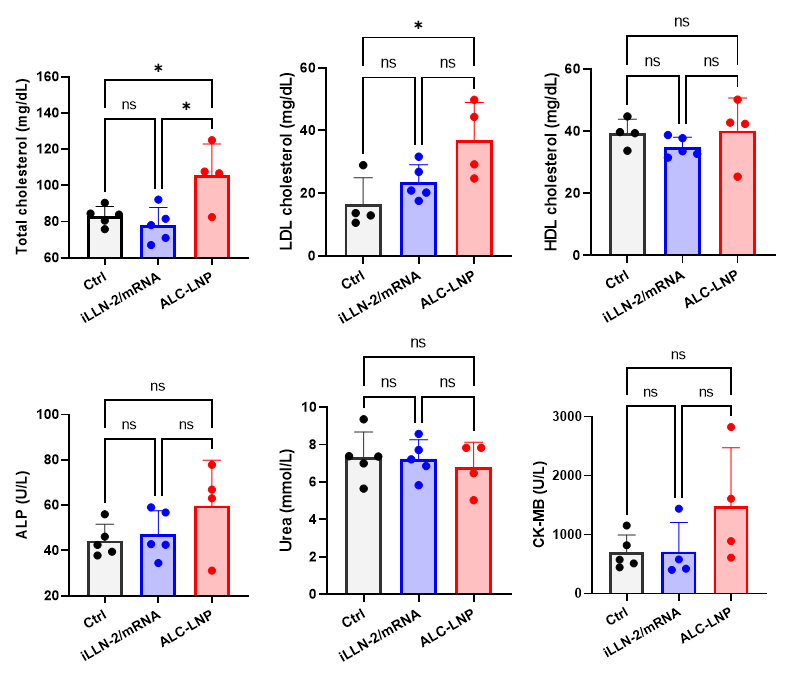


**Figure S15.** Serum levels of total cholesterol, LDL cholesterol, HDL cholesterol, ALP, urea and CK-MB on day 28 following intranasal immunization with ALC-LNP or iLLN-2/mRNA complexes (10 µg mRNA per mouse). Abbreviations: LDL, low-density lipoprotein; HDL, high-density lipoprotein; ALP, alkaline phosphatase; CK-MB, creatine kinase myocardial band. Mean ± SD (*n* = 5 for Ctrl, *n* = 5 for iLLN-2/mRNA, *n* = 4 for ALC-LNP); ^*^*P* < 0.05; ns: nonsignificant.

**Table S1.** The fluorescent signal of Cy5-mRNA in each cell area recorded for ImageJ analysis. The endosomal escape efficiency was determined by the ratio of Cy5 signal in the merged channel (mRNA escaped from endosomes) to total fluorescent signal in the Cy5-mRNA channel (internalized mRNA).

| Formulations | Cells for ImageJ analysis | Total fluorescent signal in the Cy5-mRNA channel (internalized mRNA) | Cy5 signal in the merged channel (mRNA escaped from endosomes) | Endosomal escape efficiency (%) |
| --- | --- | --- | --- | --- |
| iLLN-2/mRNA | 1 | 40.748 | 24.476 | 60.06 |
|  | 2 | 38.222 | 32.195 | 84.23 |
|  | 3 | 45.176 | 34.653 | 76.70 |
|  | 4 | 40.089 | 25.956 | 64.74 |
|  | 5 | 37.071 | 23.765 | 64.10 |
|  | 6 | 66.150 | 46.263 | 69.93 |
|  | 7 | 30.000 | 16.406 | 54.68 |
|  | 8 | 30.422 | 23.174 | 76.17 |
|  | 9 | 54.166 | 34.367 | 63.44 |
|  | 10 | 48.75 | 39.558 | 81.14 |
| ALC-LNP | 1 | 8.384 | 4.687 | 55.90 |
|  | 2 | 11.401 | 3.444 | 30.20 |
|  | 3 | 17.758 | 9.462 | 53.28 |
|  | 4 | 38.217 | 20.701 | 54.16 |
|  | 5 | 29.083 | 10.693 | 36.76 |
|  | 6 | 31.047 | 8.555 | 27.55 |
|  | 7 | 40.994 | 19.233 | 46.91 |
|  | 8 | 30.592 | 7.755 | 25.34 |
|  | 9 | 33.342 | 4.025 | 12.07 |
|  | 10 | 35.507 | 5.701 | 16.05 |

**Table S2.** The amount of lipid components of iLLN-2/mRNA and ALC-LNP formulations administered for the intranasal immunization at a dose of 10 µg mRNA per mouse.

| Formulations | Administered amount (µg per mouse) | | | | | |
| --- | --- | --- | --- | --- | --- | --- |
|  | ALC-0315 | DOTMA | Neutral lipid | Helper lipid | Triolein | PEG-lipid |
| iLLN-2/mRNA | 104.8 | 34.9 | 78.6 (DOPE) | 58.2 (*β*-sitosterol) | 8.7 | 5.8 (DSPE-PEG) |
| ALC-LNP | 135.4 | 0 | 28.4 (DSPC) | 63.0 (cholesterol) | 0 | 15.2 (ALC-0159) |
